# Risk of *Candida* Infection and Serious Infections in Patients with Moderate-to-Severe Psoriasis Receiving Biologics: A Systematic Review and Meta-Analysis of Randomized Controlled Trials

**DOI:** 10.1155/2022/2442603

**Published:** 2022-09-21

**Authors:** Yue Feng, Baosen Zhou, Zhen Wang, Guijuan Xu, Lili Wang, Tingting Zhang, Yanping Zhang

**Affiliations:** ^1^Department of Dermatology, Shenyang Seventh People's Hospital, Shenyang 110001, Liaoning, China; ^2^Department of Epidemiology, School of Public Health, China Medical University, Shenyang 110001, Liaoning, China

## Abstract

**Background:**

Biological agents used to treat moderate-to-severe plaque psoriasis have been associated with *Candida* infection and other serious infections. It is, however, necessary to verify whether biologic agents increase the risk of *Candida* infection and serious infections and whether these risks vary among biologics.

**Methods:**

PubMed, EMBASE, and Cochrane Library were searched for eligible randomized controlled trials (RCTs) from their inception to December 2021. Results from individual RCT were pooled using Peto's method with a fixed-effects model, and *I*^2^ was calculated to assess the heterogeneity. A Cochrane collaboration tool was used to examine bias risk, and Grades of Recommendation, Assessment, Development, and Evaluation (GRADE) were used to assess the quality of evidence.

**Results:**

This study included 48 published articles with data from 52 RCTs involving 27297 participants. The anti-interleukin (IL)-17 agents (95% confidence interval (CI) = 1.54–3.45, *P* < 0.0001) and anti-IL-12/23 agents (95% CI = 1.69–3.83, *P* < 0.0001) were associated with an increased risk of *Candida* infection compared with placebos, but there was no difference in *Candida* infection risk between anti-IL-17 agents and tumor necrosis factor inhibitors (TNFi) (95% CI = 0.92–3.07, *P*=0.09). There was no evidence that the biological agents increased the risk of serious infections in adult psoriasis (95% CI = 0.93–2.06, *P*=0.11) or that the biologics differed in the risk of serious infections.

**Conclusions:**

Our results indicated that anti-IL-17 agents, especially secukinumab, were associated with the increased risk of *Candida* infection. The clinically used biological agents did not increase the risk of serious infections.

## 1. Introduction

Psoriasis is a common, easy-to-relapse, chronic inflammatory skin disorder, affecting approximately 2% of the general population, with significant morbidity and a long course [1, 2]. Most patients with psoriasis suffer from chronic plaque psoriasis, which accounts for 80%–90% of all cases [3]. Psoriasis is not only manifested by cutaneous symptoms but also associated with a high risk of other diseases [4], such as psoriatic arthritis, cardiovascular disease, insulin resistance, and mental health disorders, thus causing a severe burden to patients and society [5].

Psoriasis was originally thought as a keratinocyte-driven autoinflammatory condition given the dysfunctional proliferation of keratinocytes. Nevertheless, research conducted in the 1970s identified a significant role for T cells, emphasizing their role in autoimmunity [3]. The interleukin (IL)-17/tumor necrosis factor-*α* (TNF*α*)/IL-23 axis is currently considered to be crucial in the pathogenesis of psoriasis [6]. The traditional systemic treatment of psoriasis does not significantly clear skin lesions and has many serious side effects [1]. All these drawbacks sought for biologics that target the inflammatory factors involved in the disorder. The use of biological agents including anti-inflammatory agents has revolutionized the management of psoriasis. Three main classes of biologic agents are used in routine clinical practice, including TNF inhibitors (TNFi, such as adalimumab, infliximab, and etanercept); anti-IL-12/23 agents (such as ustekinumab and guselkumab); and anti-IL-17 agents (such as secukinumab, ixekizumab, and brodalumab).

In spite of their efficacy [7, 8], these agents have been shown to increase the risk of *Candida* infection and serious infections [9, 10] due to the inhibition of the critical cytokine pathways involved in the immune system [11–15], which led to their discontinuation [16]. Patients suffering from *Candida* infections may also experience itching and pain, which negatively impact their quality of life. Based on the previous meta-analysis including randomized controlled trials (RCTs) and prospective cohort studies, neither an increased risk of serious infections nor a differential risk of serious infections was identified among the biological therapies in adults with psoriasis [17]. Moreover, clinical studies show that anti-IL-12/23 agents are associated with a lower risk of serious infections than TNFi and anti-IL-17 agents [9]. Despite this, recent clinical trials have shown contradictory results, making conclusion difficult. Consequently, there is still uncertainty regarding the risk of the use of different biologics. In spite of numerous reports of candidiasis in patients taking anti-IL-17 agents, the literature describes this complication with ambiguity. Yet, a review reported that 1.4%–13.5% of patients treated with secukinumab, 0.3%–7% with brodalumab, and 0%–3.5% with ixekizumab developed *Candida* infection [18]. To the best of our knowledge, no meta-analysis has assessed the risk of *Candida* infection in patients with psoriasis who received these biologics.

The risk of *Candida* infection and serious infections in patients receiving these biological therapies needs to be evaluated in an updated manner. Based on currently available publications, this systematic review and meta-analysis intended to review RCTs that enrolled adults with moderate-to-severe plaque psoriasis to determine the risk of *Candida* infection and serious infections among these biological therapies against placebos or conventional systemic therapeutics. When possible, the difference in infection risk between biological agents was also explored.

## 2. Methods

### 2.1. Search Strategy and Study Identification

This study followed the Preferred Reporting Items for Systematic Reviews and Meta-Analyses (PRISMA) reporting guidelines. The PubMed, EMBASE, and Cochrane Library databases were searched for all studies from their inception to December 2021. The English keywords used in this study were “Psoriasis,” “Adalimumab,” “Infliximab,” “Etanercept,” “Ustekinumab,” “Guselkumab,” “Secukinumab,” “Ixekizumab,” “Brodalumab,” and “Randomized controlled trial.” Only studies on humans published in English were considered for meta-analysis. In addition, articles from the reference list were manually searched and reviewed. We were not blinded to authors, institutions, and journals while selecting trials or extracting the data.

### 2.2. Inclusion and Exclusion Criteria

The study included in the meta-analysis should meet the following criteria: (1) patient baseline characteristics were similar (Supplementary Table 2); (2) patients were ≥18 years old and had moderate-to-severe plaque psoriasis; (3) study design was RCT; (4) the reported outcomes included *Candida* infection in patients receiving anti-IL-17 agents and other biologic agents and serious infections in patients receiving at least one licensed biological agent; (5) the control group included at least one of the following therapies: another biological agent or conventional systematic therapy or placebo.

Abstracts, letters, editorials and experts' opinions, reviews without original data, case reports, and studies without control groups were excluded from the analysis. Furthermore, the following studies were excluded: studies with a total sample size of less than 50 or a group sample size of less than 25; the percentage of patients who had psoriatic arthritis of more than 50%; and studies with no clear reported outcomes of interest.

### 2.3. Data Extraction

The titles, abstracts, and full texts of the retrieved articles were reviewed to screen out the studies that met the inclusion criteria. The data were then extracted from the eligible studies. Two investigators performed all procedures independently, and other investigators provided suggestions if disagreements occurred.

The recorded data included the trial comparisons, study features, characteristics of participants, interventions, and significant outcomes. All relevant texts, tables, and figures were also assessed for data extraction. For multiple reports from the same RCT, all data were collated into a single report. For different doses of one biological agent from the same RCT, we considered the number of participants as the number of experimental groups. Whenever treatment schemes were switched and extended in RCTs (for instance, patients who were initially treated with a biological drug and subsequently switched to another biological drug), only significant outcomes were recorded before switching.

### 2.4. Quality Assessment

The risk of bias in the included articles was assessed by two independent investigators using the Cochrane collaboration tool [19]. The following processes were assessed for bias: sequence generation, allocation concealment, blinding of participants and personnel, blinding of outcome assessors, analyzing incomplete outcome data, selective outcome reporting, and examining other potential biases. Consequently, the risk of bias was classified as high, low, or unclear.

Grades of Recommendation, Assessment, Development, and Evaluation (GRADE) were used to assess the quality of evidence using GRADEpro software (version 3.6) [20]. On the basis of study design, risk of bias, inconsistency, indirectness, precision, publication bias, and other factors, the evidence quality was ranked as high, moderate, low, or very low.

### 2.5. Statistical Analysis

The number of total participants with *Candida* infection or serious infections in each study was extracted. Peto' odds ratio (OR) provided the least biased estimates for lower simulated event rates [21]. Thus, Peto's method was used, and the 95% confidence interval (CI) was determined. The heterogeneity among studies was assessed using the chi-squared (*χ*^2^) and *I*^2^ statistics. The presence of heterogeneity was considered if *I*^2^ was >50%. If *I*^2^ was <50% and the *P* value was >0.1, it was considered that the studies had no overall heterogeneity and a fixed-effect model was used. Otherwise, a random-effect model was applied to combine the extracted data using Review Manager v.5.3 (the Cochrane collaboration). A funnel plot was used to detect the potential publication bias. There were some studies related to more than one result, so they were included in more than one forest plot. A *P* value of <0.05 was considered significant. A sensitivity analysis was performed using the Mantel–Haenszel risk difference to add robustness to the results.

## 3. Results

### 3.1. Description of Studies

**Figure 1:**
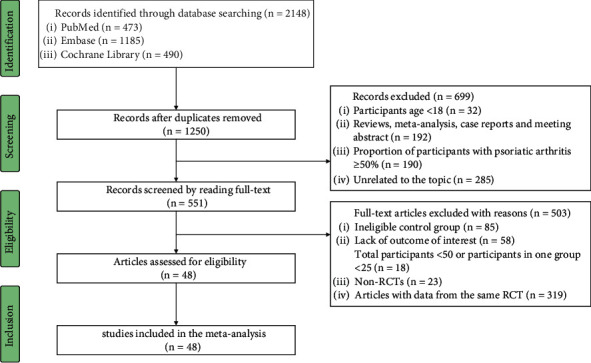
PRISMA flowchart of randomized controlled trials (RCTs) included in the analysis.

A total of 2148 published articles in English were initially identified after a systematic literature search. Finally, 48 published articles [22–69] with data from 52 RCTs involving 27297 participants were included in this meta-analysis after meticulous reviewing (Figure 1). The following information, if available, for each RCT were recorded: the first author and published year of RCTs, the registration name and number of RCTs, the countries and the clinical centers where RCTs were conducted, the length of randomized controlled phase, the basic characteristics of enrolled patients, the proportion of patients with psoriatic arthritis, the Psoriasis Area and Severity Index (PASI) score, the diagnostic criteria for moderate-to-severe psoriasis, and the recent history of serious infections (Supplementary Table 2).

There were 51 RCTs [22–63, 65–69] that reported the number of serious infections, with 10 RCTs (19.6%) [31, 32, 36, 39, 40, 43, 46, 56, 57, 66] that had no serious infection in either study arm. Eighteen RCTs (34.6%) [44, 47–55, 64, 65, 67–69] provided the number of patients who developed *Candida* infection. The randomized controlled phase lasted 12–52 weeks in all RCTs.

## 4. Meta-Analysis

### 4.1. Evidence of *Candida* Infection

In 18 RCTs [44, 47–55, 64, 65, 67–69], anti-IL-17 agents were compared with placebo, anti-IL-12/23 agents, or TNFi and the number of patients with *Candida* infection was reported. Studies that compared TNFi or anti-IL-12/23 agents with placebo were too few to determine whether they were associated with an increased risk of *Candida* infection, and therefore, they were not included in the present meta-analysis.

#### 4.1.1. Comparison of Anti-IL-17 Agents with Placebos

**Figure 2:**
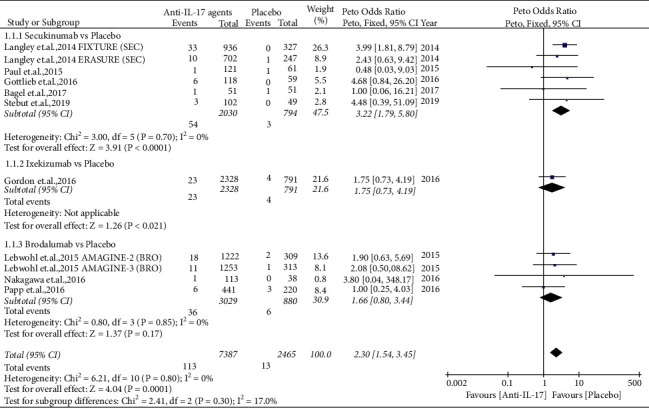
Forest plot of pooled data regarding *Candida* infections between anti-interleukin (IL)-17 agents and placebos. CI, confidence interval.

There were 11 RCTs that administrated anti-IL-17 agents and placebos [44, 48, 49, 51–55, 67] and reported a *Candida* infection rate of 1.53% in the anti-IL-17 agent arm (with a total of 7387 patients, including 2030 cases treated with secukinumab, 2328 cases treated with ixekizumab, and 3029 cases treated with brodalumab) and of 0.53% in the placebo arm (with a total of 2465 patients). The pooled Peto OR for all ten RCTs was 2.30 (95% CI = 1.54–3.45, *P* < 0.0001), showing that anti-IL-17 agents significantly increased the risk of *Candida* infection compared to placebo, with no evidence of significant heterogeneity (*I*^2^ = 0%) (Figure 2).

Furthermore, a stratified meta-analysis demonstrated that secukinumab [44, 49, 52, 55, 67] significantly increased the risk of *Candida* infection (Peto OR = 3.22, 95% CI = 1.79–5.80, *P* < 0.0001), without significant heterogeneity (*I*^2^ = 0%) (Figure 2). Only one RCT [51] compared ixekizumab with placebo and more patients who developed *Candida* infections received ixekizumab than placebo (the *Candida* infection rate was 0.99% in the ixekizumab arm and was 0.51% in the placebo arm). No significant difference in the risk of *Candida* infection between the brodalumab and placebo groups was found [48, 53, 54] (Peto OR = 1.66, 95% CI = 0.80–3.44, *P*=0.17), with no evidence of significant heterogeneity (*I*^2^ = 0%) (Figure 2).

#### 4.1.2. Comparison of Anti-IL-17 Agents with Anti-IL-12/23 Agents

**Figure 3:**
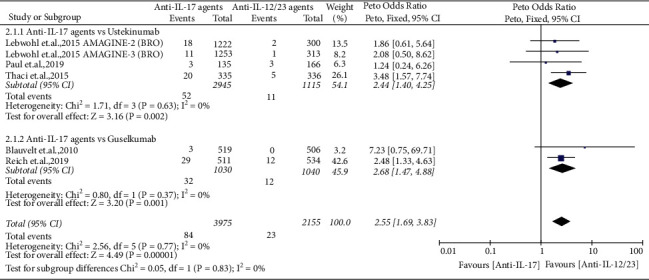
Forest plot of pooled data regarding *Candida* infections between anti-interleukin (IL)-17 agents and anti-IL-12/23 agents. CI, confidence interval.

Six RCTs [48, 50, 64, 65, 68, 69] evaluated the risk of *Candida* infection with anti-IL-17 agents (with a total of 3975 patients, including 846 cases treated with secukinumab, 654 cases treated with ixekizumab, and 2475 cases treated with brodalumab) in comparison to anti-IL-12/23 agents (with a total of 2155 patients, including 1115 cases treated with ustekinumab and 1040 cases treated with guselkumab). The reported *Candida* infection rate was 2.11% in the anti-IL-17 agent arm and 1.06% in the anti-IL-12/23 agent arm. The pooled analysis of these six RCTs indicated that anti-IL-17 agents significantly increased the risk of *Candida* infection compared to ustekinumab (Peto OR = 2.44, 95% CI = 1.40–4.25, *P*=0.002) and guselkumab (Peto OR = 2.68, 95% CI = 1.47–4.88, *P*=0.001), with no evidence of significant heterogeneity (*I*^2^ = 0%) (Figure 3).

#### 4.1.3. Comparison of Anti-IL-17 Agents with TNFi

**Figure 4:**
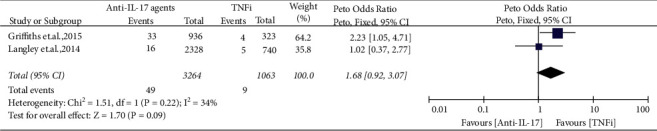
Forest plot of pooled data regarding *Candida* infections between anti-interleukin (IL)-17 agents and tumor necrosis factor inhibitors (TNFi). CI, confidence interval.

There were two RCTs [44, 47], including 3246 patients in the anti-IL-17 agent group (936 cases treated with secukinumab and 2328 cases treated with ixekizumab) and 1063 patients in the etanercept group, reporting a *Candida* infection rate of 1.50% in the anti- IL-17 agent arm and 0.85% in the etanercept arm, with no significant difference between these two arms (Peto OR = 1.68, 95% CI = 0.92–3.07, *P*=0.09) and low heterogeneity (*I*^2^ = 34%) (Figure 4).

### 4.2. Evidence of Serious Infections

#### 4.2.1. Comparison of Biologic Agents with Placebos

**Figure 5:**
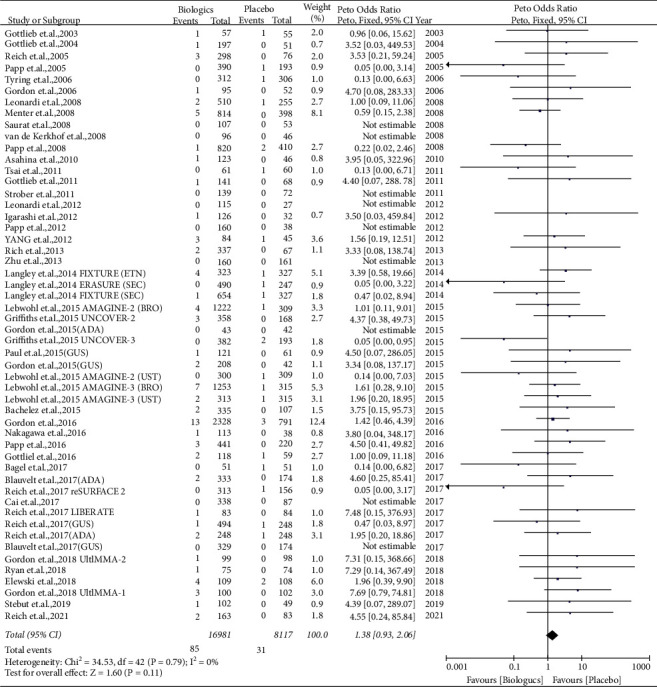
Forest plot of pooled data regarding serious infections between biological agents and placebos. CI, confidence interval.

Based on 47 RCTs using placebos and biological agents [22–33, 35–49, 51–63, 67, 69], there was a risk of serious infection rate of 0.50% (85/16981) in the biological agent arm and 0.38% (31/8117) in the placebo arm. The pooled OR for all biological agents versus placebo was 1.38 (95% CI = 0.93–2.06, *P*=0.11), with no significant difference in the risk of serious infection and no evidence of significant heterogeneity (*I*^2^ = 0%) (Figure 5).

On a subgroup analysis, there was no statistically significant difference in the risk of serious infection for patients receiving TNFi (Peto OR = 1.38, 95% CI = 0.77–2.50, *P*=0.28) [22–27, 29, 31–33, 35, 36, 41, 44–47, 56–61], anti-IL-17 agents (Peto OR = 1.41, 95% CI = 0.72–2.76, *P*=0.32) [39, 40, 42, 44, 48, 49, 51–55, 63, 67], or anti-IL-12/23 agents (Peto OR = 1.33, 95% CI = 0.55–3.23, *P*=0.53) [28, 30, 37, 38, 43, 46, 48, 56, 58, 62, 69], compared to placebo. No evidence of significant heterogeneity was found across different biological agents (*I*^2^ = 8% for ustekinumab, *I*^2^ = 37% for etanercept, and *I*^2^ = 0% for all other biologic agents) (Supplementary Figure 1).

#### 4.2.2. Comparison between Biological Agents

A total of 11 RCTs assessed the risk of serious infection between biological agents, of which two RCTs compared anti-IL-17 agents and TNFi (Peto OR = 0.99, 95% CI = 0.42–2.34, *P*=0.98) [44, 47], five RCTs compared anti-IL-17 agents and anti-IL-12/23 agents (Peto OR = 0.95, 95% CI = 0.44–2.05, *P*=0.90) [48, 50, 65, 68], and four RCTs compared anti-IL-12/23 agents and TNFi (Peto OR = 1.67, 95% CI = 0.63–4.42, *P*=0.31) [34, 46, 56, 58]. No significant difference in the risk of serious infection and no evidence of significant heterogeneity (*I*^2^ = 0% for both comparisons) were found (Supplementary Figure 2).

#### 4.2.3. Comparison of Biological Agents with Methotrexate (MTX)

Two RCTs [31, 66] assessed the difference in the risk of serious infections between biological agents and methotrexate. However, no serious infection events occurred in any of the treatment arms.

### 4.3. Risk of Bias Assessment and Sensitivity Analysis

The bias risk of the included RCTs was critically assessed using the Cochrane collaboration tool. We found that 45 RCTs (86.53%) described the methods of patient randomization, 44 RCTs (84.61%) reported concealment of allocation, 40 RCTs (76.92%) described blinding of participants and personnel, and 31 RCTs (59.61%) mentioned about an assessor of outcomes. Incomplete outcome data were well balanced in 49 RCTs (94.23%). Selective outcome reporting and other sources of bias were not identified in 38 RCTs (73.08%) and 31 RCTs (59.61%), respectively (Supplementary Table 3).

Evidence quality was ranked between high and very low using GRADEpro software. It was downgraded due to bias, imprecision, and other factors (Supplementary Table 4 and Table 5).

The sensitivity of meta-analysis using the Mantel–Haenszel methods found similar results for all comparisons.

## 5. Discussion

Although the use of biologics has improved the prognosis of patients with psoriasis, the biological agents have been associated with the risk of *Candida* infection and other serious infections [9], which led to treatment discontinuation [16]. Despite reviews of RCTs with meta-analyses and real-world data, evidence of risk remains uncertain, and whether risk varies among biological agents remains to be determined. To date, no systematic review with meta-analysis has been conducted to determine the risk of *Candida* infection in moderate-to-severe plaque psoriatic patients receiving biologics. This systematic review and meta-analysis provided an up-to-date synthesis of the published evidence relevant to the risk of *Candida* infection or serious infection related to the biologics in adult patients with moderate-to-severe plaque psoriasis.

This systematic review and meta-analysis included 48 published articles that consisted of data from 52 RCTs. The pooled analysis of 11 RCTs using placebos and anti-IL-17 agents showed that anti-IL-17 agents significantly increased the risk of *Candida* infection compared with placebos without significant heterogeneity. Previously, *Candida* infections were most commonly found in the oral cavity and vulvovaginal mucosal surfaces, with only one ear infection reported. In a separate analysis, we found that secukinumab, but not ixekizumab or brodalumab, significantly increased the risk of *Candida* infection. The incidence of *Candida* infection was 0.83%–5.08% for secukinumab, 0.99% for brodalumab, and 0.88%–1.47% for ixekizumab. Additionally, we found that anti-IL-17 agents were associated with a significantly higher risk of *Candida* infection than anti-IL-12/23 agents. There is evidence that IL-17 plays a significant role in preventing *Candida* infection in the skin and mucous membrane [70], and a higher risk of *Candida* infection is therefore expected in patients treated with anti-IL-17 agents. Furthermore, a previous meta-analysis found that patients with psoriasis had significantly higher *Candida* species colonization than controls [71]. Hence, patients with psoriasis are more likely to get *Candida* infection. In patients with psoriasis, anti-IL-17 agents, but not anti-IL-12/23 agents, may have significantly increased the risk of *Candida* infection. Although the anti-IL-17 agents increase the risk of *Candida* infection compared with the TNFi (etanercept), the difference was not statistically significant, which might have been due to the limited number of RCTs included in the analysis.

Our results highlighted the need of monitoring *Candida* infection in patients taking anti-IL-17 treatment. Therefore, we recommended that patients receiving biological agents, especially anti-IL-17, should pay closer attention to their symptoms and signs. It is important to document the sites and characteristics of the skin or mucosal lesion when *Candida* infection is highly suspected. The anti-*Candida* agents might be considered in the management of these patients. In most RCTs, the *Candida* infection was reported to be mild to moderate and anti-IL-17 agents were not discontinued, but details on how the diagnosis of *Candida* infection was made were lacking. For oropharyngeal and vulvovaginal candidiasis, the diagnosis is largely clinical. It is based on recognition of the white plaques that are typically easily scraped off and can be confirmed by a microscopic analysis using potassium hydroxide or fungal culture. However, a consensus on how to diagnose and treat *Candida* infection should be standardized for clinical application through future clinical research [72].

Although the present systematic review and meta-analysis included more recently published RCTs involving new biologics (ixekizumab, brodalumab, and guselkumab) with the large sample size, there was no significant difference between biologic agents versus placebos or among biologic agents in adult patients with moderate-to-severe psoriasis relative to serious infection risk. These outcomes were similar to the previously published review and meta-analysis [17]. Theoretically, due to the inhibition of immune system cytokine pathways during biological therapy, a higher incidence of serious infections may have been seen [11–15]. Most of the RCTs included in the meta-analysis were funded by the pharmaceutical industry in the implementation and data analysis stage but not for the writing of the manuscript and publication stage. The definition of “serious infection” was missing in most of the included articles, which may have explained the inconsistent incidence of serious infection among RCTs. More importantly, the length of the randomized controlled phase was 12–16 weeks in most RCTs (44/52 (84.62%) trials had a range from 12 to 52 weeks), and this may have led to missing of most consequences of serious infection which were previously reported to manifest 20–24 weeks after the biological therapy [73].

Nonetheless, several limitations were apparent in this meta-analysis. First, the sample size in several studies was relatively small [22, 26, 32, 37, 41, 46, 55, 59, 62, 63, 66], which may have weakened the power of our conclusion. Second, the quality of most evidence (except high-quality evidence for secukinumab versus placebo, moderate-quality evidence for anti-IL-17 agents versus anti-IL-12/23 agents for *Candida* infection, and guselkumab versus placebo for serious infections) was found to be low or very low. Third, only studies in English were included, which may therefore have resulted in selection bias. These limitations must be considered when interpreting the findings of our meta-analysis.

## 6. Conclusions

Taken together, the present study suggested that the anti-IL-17 agents, especially secukinumab, significantly increased the risk of *Candida* infection in adults with moderate-to-severe plaque psoriasis. Moreover, an increase in such risk was also found in the anti-IL-17 agents compared with the anti-IL-12/23 agents. No difference in this risk was identified between the anti-IL-17 agents and TNFi. Furthermore, there was no evidence that the biological agents increased the risk of serious infections in adult psoriasis or that the biologics differed in the risk of serious infections. However, these results should be interpreted cautiously given the limitation mentioned above. Moreover, well-designed RCTs with larger sample sizes and longer follow-ups are still needed in the future before more robust conclusions can be drawn.

## Data Availability

The randomized controlled trial data supporting this systematic review and meta-analysis are from previously reported studies and datasets, which have been cited. The processed data are available in the manuscript and the supplementary files.

## Supplementary Materials

Supplementary Materials Table 1: Full search strategy. RCT, randomized controlled trials; IL, interleukin; TNFi, tumor necrosis factor inhibitors. Table 2: Characteristics of randomized controlled trials (RCTs) included in the analysis. The basic information of the 48 included articles, which contained data from 52 RCTs with 27297 participants (the first author and published year of the article; the registration name and number of RCT; the countries and the clinical centers where RCTs were conducted; the length of randomized controlled phase; the basic characteristics of the patients who were enrolled; the proportion of patients with psoriatic arthritis; the Psoriasis Area and Severity Index (PASI) score; the criteria for moderate-to-severe psoriasis; the recent history of serious infections). Figure 1: Forest plot of pooled data of biological agents versus placebo on serious infections. There was no statistically significant difference in the risk of serious infections for patients receiving tumor necrosis factor inhibitors (TNFi), anti-interleukin (IL)-17 agents, or anti-IL-12/23 agents, compared to placebo. (a) TNFi vs. placebo; (b) anti-IL-17 agents vs. placebo; (c) anti-IL-12/23 agents vs. placebo. CI, confidence interval. Figure 2: Forest plot of pooled data of biologic agents compared to each other on serious infections. No significant difference in the risk of serious infection among different biologic agents was detected. (a) Anti-interleukin (IL)-17 agents vs. tumor necrosis factor inhibitors (TNFi); (b) anti-IL-17 agents vs. anti-IL-12/23 agents; (c) anti-IL-12/23 agents vs. TNFi. CI, confidence interval. Table 3: Risk of bias assessment for randomized controlled trials (RCTs). The risk of bias of the articles included was assessed by two independent investigators using the Cochrane collaboration tool. IL, interleukin. Table 4: Quality of evidence on *Candida* infections using Grades of Recommendations Assessment, Development, and Evaluation (GRADE). (a) Anti-interleukin (IL)-17 agents vs. placebo; (b) anti-IL-17 agents vs. anti-IL-12/23 agents; (c) anti-IL-17 agents vs. tumor necrosis factor inhibitors (TNFi). OR, odds ratio. Table 5: Quality of evidence on serious infections using Grades of Recommendations Assessment, Development, and Evaluation (GRADE). (a) Biologics vs. placebo; (b) tumor necrosis factor inhibitors (TNFi) vs. placebo; (c) anti-interleukin (IL)-17 agents vs. placebo; (d) anti-IL-12/23 agents vs. placebo; (e) anti-IL-17 agents vs. TNFi; (f) anti-IL-17 agents vs. anti-IL-12/23 agents; (g) anti-IL-12/23 agents vs. TNFi. OR, odds ratio.
