# Supplementary material for: Risk of Candida Infection and Serious Infections in Patients with Moderate-to-Severe Psoriasis Receiving Biologics: A Systematic Review and Meta-Analysis of Randomized Controlled Trials
Source: Int J Clin Pract. 2022 Sep 21;2022:2442603. doi: 10.1155/2022/2442603 (PMC9519312; doi:10.1155/2022/2442603)
Supplement: Supplementary Materials — Table 1: Full search strategy. RCT, randomized controlled trials; IL, interleukin; TNFi, tumor necrosis factor inhibitors. Table 2: Characteristics of randomized controlled trials (RCTs) included in the analysis. The basic information of the 48 included articles, which contained data from 52 RCTs with 27297 participants (the first author and published year of the article; the registration name and number of RCT; the countries and the clinical centers where RCTs were conducted; the length of randomized controlled phase; the basic characteristics of the patients who were enrolled; the proportion of patients with psoriatic arthritis; the Psoriasis Area and Severity Index (PASI) score; the criteria for moderate-to-severe psoriasis; the recent history of serious infections). Figure 1: Forest plot of pooled data of biological agents versus placebo on serious infections. There was no statistically significant difference in the risk of serious infections for patients receiving tumor necrosis factor inhibitors (TNFi), anti-interleukin (IL)-17 agents, or anti-IL-12/23 agents, compared to placebo. (a) TNFi vs. placebo; (b) anti-IL-17 agents vs. placebo; (c) anti-IL-12/23 agents vs. placebo. CI, confidence interval. Figure 2: Forest plot of pooled data of biologic agents compared to each other on serious infections. No significant difference in the risk of serious infection among different biologic agents was detected. (a) Anti-interleukin (IL)-17 agents vs. tumor necrosis factor inhibitors (TNFi); (b) anti-IL-17 agents vs. anti-IL-12/23 agents; (c) anti-IL-12/23 agents vs. TNFi. CI, confidence interval. Table 3: Risk of bias assessment for randomized controlled trials (RCTs). The risk of bias of the articles included was assessed by two independent investigators using the Cochrane collaboration tool. IL, interleukin. Table 4: Quality of evidence on Candida infections using Grades of Recommendations Assessment, Development, and Evaluation (GRADE). (a) Anti-interleukin (IL)-17 a [file 2442603.f1.pdf]

Table and Figure legends:

Table 1: Full search strategy. RCT: randomize controlled trials; IL: interleukin; TNFi: tumor necrosis factor inhibitors.

Table 2: Characteristics of randomize controlled trials (RCTs) included in the analysis. The basic information of the 48 included articles, which contained data from 52 RCTs with 27297 participants (the first author and published year of the article; the registration name and number of RCT; the countries and the clinical centers where RCTs were conducted; the the length of randomized controlled phase; the basic characteristics of the patients who were enrolled; the proportion of patients with psoriatic arthritis; the Psoriasis Area and Severity Index [PASI] score; the criteria for moderate-to-severe psoriasis; the recent history of serious infections).

Figure 1: Forest plot of pooled data of biological agents versus placebo on serious infections. There was no statistically significant difference in the risk of serious infections for patients receiving tumor necrosis factor inhibitors (TNFi), anti-interleukin (IL)-17 agents, or anti-IL-12/23 agents, compared to placebo. (a) TNFi vs. Placebo; (b) Anti-IL-17 agents vs. Placebo; (c) Anti-IL-12/23 agents vs. Placebo. CI: confidence interval.

Figure 2: Forest plot of pooled data of biologic agents comparing to each other on serious infections. No significant difference in the risk of serious infection among different biologic agents was detected. (a) Anti-interleukin (IL)-17 agents vs. tumor necrosis factor inhibitors (TNFi); (b) Anti-IL-17 agents vs. Anti-IL-12/23 agents; (c) Anti-IL-12/23 agents vs. TNFi. CI: confidence interval.

Table 3: Risk of bias assessment for randomize controlled trials (RCTs). The risk of bias of the articles included was assessed by two independent investigators using the Cochrane collaboration tool. IL: interleukin.

Table 4: Quality of evidence on *Candid*a infections using Grades of Recommendations Assessment, Development and Evaluation (GRADE). (a) Anti-interleukin (IL)-17 agents vs. Placebo; (b) Anti-IL-17 agents vs. Anti-IL-12/23 agents; (c) Anti-IL-17 agents vs. tumor necrosis factor inhibitors (TNFi). OR: odds ratio.

Table 5: Quality of evidence on serious infections using Grades of Recommendations Assessment, Development and Evaluation (GRADE). (a) Biologics vs. Placebo; (b) tumor necrosis factor inhibitors (TNFi) vs. Placebo; (c) Anti-interleukin (IL)-17 agents vs. Placebo; (d) Anti-IL-12/23 agents vs. Placebo; (e) Anti-IL-17 agents vs. TNFi; (f) Anti-IL-17 agents vs. Anti-IL-12/23 agents; (g) Anti-IL-12/23 agents vs. TNFi. OR: odds ratio.

Supplementary files

Table 1: Full search strategy. RCT: randomize controlled trials; IL: interleukin; TNFi: tumor necrosis factor inhibitors.

PubMed (31-12-2021)

Limit: Randomized Controlled Trial only

Concepts	#	Search strategy	Results
**Psoriasis**	1	((psoriasis[Title/Abstract]) OR (Psoriases[Title/Abstract]) OR (Pustulosis of Palms and Soles[Title/Abstract]) OR (Pustulosis Palmaris et Plantaris[Title/Abstract]) OR (Palmoplantaris Pustulosis[Title/Abstract]) OR (Pustular Psoriasis of Palms and Soles[Title/Abstract]))	
**TNFi combined**	2	(((infliximab[Title/Abstract]) OR (Monoclonal Antibody cA2[Title/Abstract]) OR (cA2, Monoclonal Antibody[Title/Abstract]) OR (MAb cA2[Title/Abstract]) OR (Infliximab-abda[Title/Abstract]) OR (Renflexis[Title/Abstract]) OR (Infliximab-dyyb[Title/Abstract]) OR (Inflectra[Title/Abstract]) OR (Remicade[Title/Abstract])) OR ((etanercept[Title/Abstract]) OR (TNFR-Fc Fusion Protein[Title/Abstract]) OR (Fusion Protein, TNFR-Fc[Title/Abstract]) OR (TNFR Fc Fusion Protein[Title/Abstract]) OR (TNR 001[Title/Abstract]) OR (TNT Receptor Fusion Protein[Title/Abstract]) OR (TNTR-Fc[Title/Abstract]) OR (TNR-001[Title/Abstract]) OR (TNR001[Title/Abstract]) OR (Etanercept-szzs[Title/Abstract]) OR (TNF Receptor Type II-IgG Fusion Protein[Title/Abstract]) OR (TNF Receptor Type II IgG Fusion Protein[Title/Abstract]) OR (Erelzi[Title/Abstract]) OR (Recombinant Human Dimeric TNF Receptor Type II-IgG Fusion Protein[Title/Abstract]) OR (Recombinant Human Dimeric TNF Receptor Type II IgG Fusion Protein[Title/Abstract]) OR (Enbrel[Title/Abstract])) OR ((adalimumab[Title/Abstract]) OR (Humira[Title/Abstract]) OR (Adalimumab-adbm[Title/Abstract]) OR (Amjevita[Title/Abstract]) OR (Adalimumab-atto[Title/Abstract]) OR (Cyltezo[Title/Abstract]) OR (D2E7 Antibody[Title/Abstract]) OR (Antibody, D2E7[Title/Abstract])))	
**anti-IL-17 agents combined**	2	(((Secukinumab[Title/Abstract]) OR (Cosentyx[Title/Abstract]) OR (AIN 457[Title/Abstract]) OR (AIN457[Title/Abstract]) OR (AIN-457[Title/Abstract])) OR ((Ixekizumab[Title/Abstract]) OR (Taltz[Title/Abstract]) OR (LY2439821[Title/Abstract]) OR (LY-2439821[Title/Abstract])) OR ((Brodalumab[Title/Abstract]) OR (Siliq[Title/Abstract]) OR (KHK-4827[Title/Abstract]) OR (KHK4827[Title/Abstract]) OR (AMG-827[Title/Abstract]) OR (AMG 827[Title/Abstract])))	
**anti-IL-12/23 agents combined**	3	(((guselkumab[Title/Abstract]) OR (Tremfya[Title/Abstract]) OR (CNTO 1959[Title/Abstract]) OR (CNTO-1959[Title/Abstract])) OR ((Ustekinumab[Title/Abstract]) OR (Stelara[Title/Abstract]) OR (CNTO 1275[Title/Abstract]) OR (CNTO-1275[Title/Abstract])))	
**Total**	4	#1 AND (#2 OR #3 OR #4)	**473**

Embase.com (31-12-2021)

Limit: Randomized Controlled Trial only

Concepts		#	Search strategy	Results
psoriasis		1	'psoriases’:ab,ti	
		2	‘psoriases’:ab,ti	
		3	‘pustulosis of Palms and Soles’:ab,ti	
		4	‘pustulosis Palmaris et Plantaris’:ab,ti	
		5	‘Palmoplantaris Pustulosis’:ab,ti	
		6	‘pustular Psoriasis of Palms and Soles’:ab,ti	
**Psoriasis combined**		**7**	**#1 OR #2 OR #3 OR #4 OR #5 OR #6**	
TNFi	infliximab	8	‘Infliximab’:ab,ti	
		9	‘Monoclonal Antibody cA2’:ab,ti	
		10	‘cA2, Monoclonal Antibody’:ab,ti	
		11	‘MAb cA2’:ab,ti	
		12	‘Infliximab-abda’:ab,ti	
		13	‘Renflexis’:ab,ti	
		14	‘Infliximab-dyyb’:ab,ti	
		15	‘Inflectra’:ab,ti	
		16	‘Remicade’:ab,ti	
	infliximab combined	17	#8 OR #9 OR #10 OR #11 OR #12 OR #13 OR #14 OR #15 OR #16	
	etanercept	18	‘etanercept’:ab,ti	
		19	‘TNFR-Fc Fusion Protein’:ab,ti	
		20	‘Fusion Protein, TNFR-Fc’:ab,ti	
		21	‘TNFR Fc Fusion Protein’:ab,ti	
		22	‘TNR 001’:ab,ti	
		23	‘TNT Receptor Fusion Protein’:ab,ti	
		24	‘TNTR-Fc’:ab,ti	
		25	‘TNR-001’:ab,ti	
		26	‘TNR001’:ab,ti	
		27	‘Etanercept-szzs’:ab,ti	
		28	‘TNF Receptor Type II-IgG Fusion Protein’:ab,ti	
		29	‘TNF Receptor Type II IgG Fusion Protein’:ab,ti	
		30	‘Erelzi’:ab,ti	
		31	‘Recombinant Human Dimeric TNF Receptor Type II-IgG Fusion Protein’:ab,ti	
		32	‘Recombinant Human Dimeric TNF Receptor Type II IgG Fusion Protein’:ab,ti	
		33	‘Enbrel’:ab,ti	
	etanercept combined	34	#18 OR #19 OR #20 OR #21 OR #22 OR #23 OR #24 OR #25 OR #26 OR #27 OR #28 OR #29 OR #30 OR #31 OR #32 OR #33	

TNFi	adalimumab	35	'Adalimumab':ab,ti	
		36	'Humira':ab,ti	
		37	'Adalimumab-adbm':ab,ti	
		38	'Amjevita':ab,ti	
		39	'Adalimumab-atto':ab,ti	
		40	'Cyltezo':ab,ti	
		41	'D2E7 Antibody':ab,ti	
		42	'Antibody, D2E7':ab,ti	
	adalimumab combined	43	#35 OR #36 OR #37 OR #38 OR #39 OR #40 OR #41 OR #42	
**TNFi combined**		**44**	**#17 OR #34 OR #43**	
anti-IL-17 agents	Secukinumab	45	'Secukinumab':ab,ti	
		46	'Cosentyx':ab,ti	
		47	'AIN 457':ab,ti	
		48	'AIN457':ab,ti	
		49	'AIN-457':ab,ti	
	Secukinumab combined	50	#45 OR #46 OR #47 OR #48 OR #49	
	Ixekizumab	51	'Ixekizumab':ab,ti	
		52	'Taltz':ab,ti	
		53	'LY2439821':ab,ti	
		54	'LY-2439821':ab,ti	
	Ixekizumab combined	55	#51 OR #52 OR #53 OR #54	
	Brodalumab	56	'Brodalumab':ab,ti	
		57	'Siliq':ab,ti	
		58	'KHK-4827':ab,ti	
		59	'KHK4827':ab,ti	
		60	'AMG-827':ab,ti	
		61	'AMG 827':ab,ti	
	Brodalumab combined	62	#56 OR #57 OR #58 OR #59 OR #60 OR #61	
**anti-IL-17 agents combined**		**63**	**#50 OR #55 OR #62**	
anti-IL-12/23 agents	Ustekinumab	64	'Ustekinumab':ab,ti	
		65	'Stelara':ab,ti	
		66	'CNTO 1275':ab,ti	
		67	'CNTO-1275':ab,ti	
	Ustekinumab combined	68	#64 OR #65 OR #66 OR #67	
	guselkumab	69	'guselkumab ':ab,ti	
		70	'tremfya':ab,ti	
		71	'CNTO 1959':ab,ti	
		72	'CNTO-1959 ':ab,ti	
	guselkumab combined	73	#69 OR #70 OR #71 OR #72	
**anti-IL-12/23 agents combined**		**74**	**#68 OR #73**	
**Total**		**75**	**#7 AND (#44 OR #63 OR #74)**	**1185**

Cochrane Library(31-12-2021)

Limit: trials only

Concepts		#	Search strategy	Results
psoriasis		1	(psoriases):ab,ti	
		2	(psoriases):ab,ti	
		3	(pustulosis of Palms and Soles):ab,ti	
		4	(pustulosis Palmaris et Plantaris):ab,ti	
		5	(Palmoplantaris Pustulosis):ab,ti	
		6	(pustular Psoriasis of Palms and Soles):ab,ti	
**Psoriasis combined**		**7**	**#1 OR #2 OR #3 OR #4 OR #5 OR #6**	**8575**
TNFi	infliximab	8	(Infliximab):ab,ti	
		9	(Monoclonal Antibody cA2):ab,ti	
		10	(cA2, Monoclonal Antibody):ab,ti	
		11	(MAb cA2):ab,ti	
		12	(Infliximab-abda):ab,ti	
		13	(Renflexis):ab,ti	
		14	(Infliximab-dyyb):ab,ti	
		15	(Inflectra):ab,ti	
		16	(Remicade):ab,ti	
	infliximab combined	17	#8 OR #9 OR #10 OR #11 OR #12 OR #13 OR #14 OR #15 OR #16	
	etanercept	18	(etanercept):ab,ti	
		19	(TNFR-Fc Fusion Protein):ab,ti	
		20	(Fusion Protein, TNFR-Fc):ab,ti	
		21	(TNFR Fc Fusion Protein):ab,ti	
		22	(TNR 001):ab,ti	
		23	(TNT Receptor Fusion Protein):ab,ti	
		24	(TNTR-Fc):ab,ti	
		25	(TNR-001):ab,ti	
		26	(TNR001):ab,ti	
		27	(Etanercept-szzs):ab,ti	
		28	(TNF Receptor Type II-IgG Fusion Protein):ab,ti	
		29	(TNF Receptor Type II IgG Fusion Protein):ab,ti	
		30	(Erelzi):ab,ti	
		31	(Recombinant Human Dimeric TNF Receptor Type II-IgG Fusion Protein):ab,ti	
		32	(Recombinant Human Dimeric TNF Receptor Type II IgG Fusion Protein):ab,ti	
		33	(Enbrel):ab,ti	
	etanercept combined	34	#18 OR #19 OR #20 OR #21 OR #22 OR #23 OR #24 OR #25 OR #26 OR #27 OR #28 OR #29 OR #30 OR #31 OR #32 OR #33	

TNFi	adalimumab	35	(Adalimumab):ab,ti	
		36	(Humira):ab,ti	
		37	(Adalimumab-adbm):ab,ti	
		38	(Amjevita):ab,ti	
		39	(Adalimumab-atto):ab,ti	
		40	(Cyltezo):ab,ti	
		41	(D2E7 Antibody):ab,ti	
		42	(Antibody, D2E7):ab,ti	
	adalimumab combined	43	#35 OR #36 OR #37 OR #38 OR #39 OR #40 OR #41 OR #42	
**TNFi combined**		**44**	**#17 OR #34 OR #43**	**6946**
anti-IL-17 agents	Secukinumab	45	(Secukinumab):ab,ti	
		46	(Cosentyx):ab,ti	
		47	(AIN 457):ab,ti	
		48	(AIN457):ab,ti	
		49	(AIN-457):ab,ti	
	Secukinumab combined	50	#45 OR #46 OR #47 OR #48 OR #49	
	Ixekizumab	51	(Ixekizumab):ab,ti	
		52	(Taltz):ab,ti	
		53	(LY2439821):ab,ti	
		54	(LY-2439821):ab,ti	
	Ixekizumab combined	55	#51 OR #52 OR #53 OR #54	
	Brodalumab	56	(Brodalumab):ab,ti	
		57	(Siliq):ab,ti	
		58	(KHK-4827):ab,ti	
		59	(KHK4827):ab,ti	
		60	(AMG-827):ab,ti	
		61	(AMG 827):ab,ti	
	Brodalumab combined	62	#56 OR #57 OR #58 OR #59 OR #60 OR #61	
**anti-IL-17 agents combined**		**63**	**#50 OR #55 OR #62**	**1711**
anti-IL-12/23 agents	Ustekinumab	64	(Ustekinumab):ab,ti	
		65	(Stelara):ab,ti	
		66	(CNTO 1275):ab,ti	
		67	(CNTO-1275):ab,ti	
	Ustekinumab combined	68	#64 OR #65 OR #66 OR #67	
	guselkumab	69	(guselkumab):ab,ti	
		70	(tremfya):ab,ti	
		71	(CNTO 1959):ab,ti	
		72	(CNTO-1959):ab,ti	
	guselkumab combined	73	#69 OR #70 OR #71 OR #72	
**anti-IL-12/23 agents combined**		**74**	**#68 OR #73**	**1284**

RCT	75	(Randomized Controlled Trial):pt	
	76	(Equivalence Trial):pt	
	77	(Pragmatic Clinical Trial):pt	
**RCT combined**	**78**	**#75 OR #76 OR #77**	
**Total**	**75**	**(#44 OR #63 OR #74) AND #7 AND #78**	**490**

Table 2. Characteristics of randomize controlled trials (RCTs) included in the analysis.

study	the registration name or number	No. of study sites and countries	Randomized controlled-phase (weeks)	Interventions of treatment group	No. of participants receiving treatment	Mean age ± SD (median) years	Proportion of patients with psoriatic arthritis (%)	Mean Psoriasis Area and Severity Index Score ± SD (median)	Criteria for the moderate-to-severe psoriasis[1]	whether the recent history of serious infection was excluded
**Secukinumab vs. Placebo**										
Rich et.al.,2013	(NCT00941031)	60 sites (2009.07-2010.12)	12w	150mg-single(0w); monthly(0w,4w,8w) ; early(0w,1w,2w,4w)	single:66; monthly:138; early:133	single:42.7±11.32; monthly:44.2±12.96; early:44.5 ± 12.45; PBO 44.2 ±12.59	single:22.7; monthly:32.6; early:29.3; PBO:17.9	single:19.9±6.73; monthly:20.8±8.08; early:19.9 ± 7.81; PBO:20.5 ± 9.31	BSA≥10% and IGA≥3 and PASI≥12	yes
Langley et.al.,2014	ERASURE (NCT01365455)	231 sites (2011.06-2013.06)	12w	150mg;300mg. (1w, 2w, 3w, 4w,q4w)	150mg: 245; 300mg:245	150mg: 44.9±13.3; 300mg:44.9±13.5; PBO 45.4±12.6	150mg: 18.8; 300mg:23.3; PBO 27.4	150mg: 22.3±9.8; 300mg:22.5±9.2; PBO 21.4±9.1	BSA≥10% and IGA≥3 and PASI≥12	yes(within 2 weeks)
Langley et.al.,2014	FIXTURE (NCT01358578)	231 sites (2011.06-2013.06)	12w	150mg;300mg. (1w, 2w, 3w, 4w,q4w)	150mg: 327; 300mg:326	150mg: 45.4±12.9; 300mg:44.5±13.2; PBO 44.1±12.6	150mg: 15.0; 300mg:15.3; PBO 15.0	150mg: 23.7±10.5; 300mg:23.9±9.9; PBO 24.1±10.5	BSA≥10% and IGA≥3 and PASI≥12	yes(within 2 weeks)
Paul et.al.,2015	JUNCTURE (NCT01636687)	38 sites (2012.10-2013.04)	12w	150mg;300mg. (0w, 1w, 2w, 3w,q4w)	150mg: 61; 300mg:60	150mg: 43.9±14.41; 300mg:46.6±14.23; PBO 43.7±12.74	150mg: 26.2; 300mg:23.3; PBO 19.7	150mg: 22.0±8.85; 300mg:18.9±6.37; PBO 19.4±6.70	BSA≥10% and IGA≥3 and PASI≥12	not reported
Gottlieb et.al.,2016	FEATURE (NCT01555125)	32 site (2012.05-2013.10)	12w	150mg;300mg. (0w, 1w, 2w, 3w,q4w)	150mg: 59; 300mg:59	150mg: 46.0±15.09; 300mg:45.1±12.57; PBO 46.5±14.14	not reported	150mg: 21.4±9.1; 300mg:20.7±7.95; PBO 21.1±8.49	BSA≥10% and IGA≥3 and PASI≥12	yes(within 2 weeks)
Bagel et.al.,2017	SCALP (NCT02267135)	17 sites (2014.09-2015.12)	12w	300 mg (1w, 2w, 3w, q4w)	51	SEC:42.7 ±13.4; PBO:41.1 ±14.2	not reported	SEC:8.4 ±7.6; PBO:10.1 ±7.8	PSSI≥12, with ≥30% of the scalp surface area affected, IGAI≥3	no
von Stebut et.al.,2019	CARIMA (NCT02559622)	23 sites (2014.04-2016.04)	12w	150mg;300mg. (1w, 2w, 3w, 4w,q4w)	150mg: 54; 300mg:48	150mg: 46.0±14.4; 300mg:44.2±12.9; PBO 45.2±12.2	150mg: 27.8; 300mg:25.0; PBO 16.3	150mg: 21.7±10.5; 300mg:19.3±7.9; PBO 18.4±5.2	PASI≥ 10	yes
**Ixekizumab vs. Placebo**										
Leonardi et.al.,2012	(NCT01107457)	(2010.04-2011.03)	20w	10 mg;25mg;75 mg; 150 mg (0w, 2w, 4w, q4w)	10 mg:28; 25 mg:30; 75mg:29; 150 mg:28	10 mg:48±11; 25 mg:46±15; 75mg:46±13; 150mg:46±13; PBO:45±13	10 mg:25.0; 25 mg:36.7; 75mg:27.6; 150mg:28.6; PBO:14.8	10mg:19.2±8.0; 25mg:18.6±4.9; 75mg:17.2±4.3; 150mg:17.7±6.2; PBO:16.5±5.3	BSA≥10% and PGA≥3 and PASI≥12	yes(recent)
Gordon et.al.,2016	UNCOVER-1 (NCT01474512)	/	12w	160 mg(0w)-80 mg(eow or q4w)	eow:433; q4w:432	eow:45.0±120.; q4w:46.0±13.0; PBO 46.0±13.0	not reported	eow:20.0±8.0; q4w:20.0±7.0; PBO 20.0±9.0	BSA≥10% and PGA≥3 and PASI≥12	yes

Gordon et.al.,2016	UNCOVER-2 (NCT01597245)	(2012.05-2013.12)	12w	160 mg(0w)-80 mg(eow or q4w)	eow:351; q4w:347	eow:45.0±13.0; q4w:45.0±14.0; PBO 45.0±12.0	not reported	eow:19.0±7.0; q4w:20.0±7.0; PBO 21.0±8.0	BSA≥10% and PGA≥3 and PASI≥12	yes
Gordon et.al.,2016	UNCOVER-3 (NCT01646177)	(2012.08-2014.02)	12w	160 mg(0w)-80 mg(eow or q4w)	eow:385; q4w:386	eow:46.0±13.0; q4w:46±13.0; PBO 46±12.0	not reported	eow:21±8.0; q4w:21±8.0; PBO 21±8.0	BSA≥10% and PGA≥3 and PASI≥12	yes
Ryan et.al.,2018	IXORA-Q (NCT02718898)	34 sites (2016.05-2016.12)	12w	160mg(0w) – 80 mg(eow)	75	IXE:43.1±13.0; PBO:44.4 ±12.6	not reported	IXE:26.3±15.4; PBO:28.3±14.4	sPGA-G ≥ 3 and sPGA ≥ 3	yes(12w)
**Brodalumab vs. Placebo**										
Papp et.al.,2012	(NCT00975637)	23 sites (2009.12-2010.04)	12w	70mg;140mg;210mg (0w,1w, 2w,eow) ; 280 mg(0w,q4w)	160	BRO:42.6±11.7; PBO:41.8±14.4	BRO:24.0; PBO:18.0	BRO:19.2±6.8; PBO:18.9±5.9	BSA≥10% and PASI≥12	yes(recent)
Lebwohl et.al.,2015	AMAGINE-2 (NCT01708603)	142 sites (2012.08-2014.09)	12w	140 mg;210 mg (0w,1w, 2w,eow)	140mg: 610; 210mg:612	140mg:45.0±13.0; 210mg:45.0±13.0; PBO 44.0±13.0	140mg: 21.0; 210mg:19.0 PBO 17.0	140mg:20.5±8.2; 210mg:20.3±8.3 PBO 20.4±8.2	BSA≥10% and sPGA≥3 and PASI≥12	not reported
Lebwohl et.al.,2015	AMAGINE-3 (NCT01708629)	142 sites (2012.09-2014.08)	12w	140mg;210mg (0w,1w, 2w,eow)	140mg: 629; 210mg:624	140mg: 45.0±13.0; 210mg:45.0±13.0; PBO 44.0±13.0	140mg: 21.0; 210mg:20.0 PBO 19.0	140mg:20.1±8.5; 210mg:20.4±8.3 PBO 20.1±8.7	BSA≥10% and sPGA≥3 and PASI≥12	not reported
Nakagawa et.al.,2016	(NCT01748539)	multicenter	12w	70mg;140mg;210mg (0w,1w, 2w,eow)	70mg:39; 140mg: 37; 210mg:37	70mg:43.4±11.8; 140mg:46.4±13.2; 210mg:46.4±11.8; PBO 46.6±10.8	70mg:15.4; 140mg: 16.2; 210mg:13.5; PBO 18.4	70mg:27.58±11.61; 140mg:28.53±10.77; 210mg:27.98±14.35; PBO 23.97±8.90	BSA≥10% and PASI≥12	yes(recent)
Papp et.al.,2016	AMAGINE-1 (NCT01708590)	73 centres (2012.08-2014.03)	12w	140 mg;210mg (0w,1w,2w,eow)	140mg: 219; 210mg:222	140mg:46.0±13.0; 210mg:46.0±12.0; PBO 47.0±13.0	140mg: 27.0; 210mg:26.0; PBO 29.0	140mg:20.0±7.4; 210mg:19.4±6.6 PBO 19.7±7.7	BSA≥10% and sPGA≥3 and PASI≥12	yes(within 8weeks)
**Adalimumab vs. Placebo**										
Gordon et.al.,2006	/	18 sites	12w	80mg(0w)-40mg(eow); 80mg(0w,1w)- 40mg(weekly)	eow 45; weekly 50	eow 46; weekly 44; PBO 43	eow 33; weekly 24; PBO 31	eow 16.7; weekly 14.5; PBO 16	BSA≥5%	no
Saurat et.al.,2008	CHAMPION (NCT00235820)	28 sites	16w	80mg(0w)-40mg(eow)	108	ADA 42.9± 12.6 PBO 40.7± 11.4	ADA 21.3; PBO 20.8	ADA 20.2± 7.5; PBO 19.2± 6.9	BSA≥10% and PASI≥10	not reported
Menter et.al.,2008	REVEAL (NCT00237887)	81 sites	16w	80mg(0w)-40mg(eow)	814	ADA 44.1± 13.2 PBO 45.4± 13.4	ADA 27.5 PBO 28.4	ADA 19.0± 7.08 PBO 18.8± 7.09	BSA≥10% and PASI≥12 and PGA ≥3	not reported
Asahina et.al.,2010	/	42 sites (2005.09-2006.12)	24w	40 mg(eow); 80mg(0w)-40mg(eow); 80 mg(eow)	123	40mg(47.8±12.8); 80-40mg (44.2±14.32); 80mg(43.5±12.40); PBO(43.9±10.75)	not reported	40mg(25.44±8.98); 80-40mg (30.24±10.95); 80mg(28.27±11.03); PBO(29.10±11.77)	BSA≥10% and PASI≥12	not reported

Gordon et.al.,2015	X-PLORE (NCT01483599)	43 sites (2011.10-2013.08)	16w	80mg(0w)-40mg(eow)	43	ADA 50.0 PBO 46.5	ADA 25.6 PBO 28.6	ADA 20.2± 7.58 PBO 21.8± 9.98	BSA≥10% and PGA≥3 and PASI≥12	not reported
Blauvelt et.al.,2017	VOYAGE 1 (NCT02207231)	101 sites (2014.12-2016.04)	16w	80mg(0w)-40mg(eow)	333	ADA 42.9±12.58 PBO 44.9±12.90	ADA 18.6 PBO 17.2	ADA 22.4±8.97 PBO 20.4±8.74	BSA≥10% and IGA≥3 and PASI≥12	not reported
Reich et.al.,2017	VOYAGE 2 (NCT02207244)	115 sites (2014.11-2016.05)	16w	80mg(0w)-40mg(eow)	248	ADA 43.2±11.9 PBO 43.3±12.4	ADA 17.7 PBO 18.5	ADA 21.7±9.0 PBO 21.5 ±8.0	BSA≥10% and IGA≥3 and PASI≥12	not reported
Cai et.al.,2017	(NCT01646073)	16 sites (2012.08-2013.12)	12w	80mg(0w)-41mg(eow)	338	ADA 43.1±11.91 PBO 43.8±12.45	ADA 12.7 PBO 11.5	ADA 28.2±12.0 PBO 25.6±10.98	BSA≥10% and PGA≥3 and PASI≥10	not reported
Elewski et.al.,2018	(NCT02016482)	32 sites	16w	80mg(0w)-40mg(eow)	109	ADA 47.2±11.86 PBO 46.2±12.13	ADA 27.5 PBO 29.6	ADA 12.3±8.59 PBO 12.8±9.43	BSA≥10% or BSA≥5% with a total mNAPSI≥20 and target fingernail mNAPSI≥8, PGA-F and PGA-S at least moderate severity, NPPFS≥3 or NRS≥3	not reported
**Infliximab vs. Placebo**										
Gottlieb et.al.,2004	SPIRIT	24 sites (2001-2003)	30w	3 mg/kg;5 mg/kg (0w,2w,6w)-additional 3 mg/kg;5 mg/kg (at 26w according to sPGA)	197	INF 44.0(35,53) PBO 45.0(30,52)	INF 31.3 PBO 33.3	INF 19.0(15,27) PBO 18.0(15,27)	BSA≥10% and PASI≥12	yes(within 2 months)
Reich et.al.,2005	EXPRESS	32 sites	12w	5 mg/kg (0w, 2w, 6w and q8w)	298	INF 42.6±11.7 PBO 43.8±12.6	INF 31.0 PBO 29.0	INF 22.9±9.3 PBO 22.8±8.7	BSA≥10% and PASI≥12	yes
YANG et.al.,2012	/	9 sites (2009.02-2010.02)	12w	5 mg/kg (0w, 2w, and 6w)	84	INF 39.4±12.3 PBO 40.1±11.1	not reported	INF 23.9±10.7 PBO 25.3±12.7	BSA≥10% and PASI≥12	yes(within 2 months)
**Etanercept vs. Placebo**										
Gottlieb et.al.,2003	/	multicenter	24w	25mg(biw)	57	ETN 48.2(25-72) PBO 46.5(18-77)	ETN 28 PBO 35	ETN 17.8±1.1 PBO 19.5±1.3	BSA≥10%	not reported
Papp et.al.,2005	/	50 sites	12w	25mg(biw); 50 mg(biw)	25mg196; 50 mg194	25 mg 46(20-87); 50 mg 44.5 (21-80); PBO 44(18-80)	25 mg 26; 50 mg 28; PBO 26	25 mg 16.9(4.0-51.2); 50 mg 16.1(7.0-57.3); PBO 16(7.0-62.4)	BSA≥10% and PASI≥10 at screening	yes(within 1 months)
Tyring et.al.,2006	(NCT00111449)	39 sites (2003.06-2004.01)	12w	50mg(biw)	312	ETN 45.8±12.8 PBO 45.6±12.1	ETN 35 PBO 33	ETN 18.3±7.6 PBO 18.1±7.4	BSA≥10% and PASI≥10 at screening	not reported

van de Kerkhof et.al.,2008	/	(2006.06-2007.05)	12w	50 mg(qw)	96	ETN 45.9 ± 12.8<br>PBO 43.6 ± 12.6	ETN 15.6<br>PBO 10.9	ETN 21.4 ± 9.3<br>PBO 21.0 ± 8.7	BSA≥10% and PASI≥10 at screening	yes(within 1 months)
Gottlieb et.al.,2011	(NCT00691964)	33 sites<br>(2008.06-2009.03)	12w	50 mg(biw)	141	ETN 43.1 ± 12.5<br>PBO 44.0 ± 13.6	ETN 22.7<br>PBO 20.6	ETN 19.4 ± 8.0<br>PBO 18.5 ± 6.9	BSA≥10% and PGA≥3 and PASI≥12	not reported
Strober et.al.,2011	(NCT00710580)	41 sites<br>(2008.07-2009.04)	12w	50 mg(biw)	139	ETN 45.2 ± 14.8<br>PBO 45.0 ± 13.9	ETN 33.1<br>PBO 20.8	ETN 18.5 ± 6.0<br>PBO 18.3 ± 6.4	BSA≥10% and PGA≥3 and PASI≥12	not reported
Langley et.al.,2014	FIXTURE<br>(NCT01358578)	231 sites<br>(2011.06-2013.06)	12w	50 mg(biw)	323	ETN 43.8±13.0<br>PBO 44.1±12.6	ETN 13.5<br>PBO 15.0	ETN 23.2±9.8<br>PBO 24.1±10.5	BSA≥10% and IGA≥3 and PASI≥12	yes(within 2 weeks)
Griffiths et.al.,2015	UNCOVER-2<br>(NCT01597245)	(2012.05-2013.12)	12w	50 mg(biw)	358	ETN 45.0±13.0<br>PBO 45.0±12.0	not reported	ETN 19.0±7.0<br>PBO 21.0±8.0	BSA≥10% and PGA≥3 and PASI≥12	not reported
Griffiths et.al.,2015	UNCOVER-3<br>(NCT01646177)	(2012.08-2014.02)	12w	50 mg(biw)	382	ETN 46.0±14.0<br>PBO 46.0±12.0	not reported	ETN 21±8.0<br>PBO 21±8.0	BSA≥10% and PGA≥3 and PASI≥12	not reported
Bachelez et.al.,2015	(NCT01241591)	122 sites	12w	50 mg(biw)	335	ETN 42.0(18.0-74.0)<br>PBO 46.0(21.0-81.0)	ETN 21.0<br>PBO 24.0	ETN 19.4(12.0-63.6)<br>PBO 19.5(12.4-54.6)	BSA≥10% and PGA≥3 and PASI≥12	yes(within 6 months)
Reich et.al.,2017	LIBERATE<br>(NCT01690299)	multicenter	16w	50 mg(qw)	83	ETN 47.0±14.1<br>PBO 43.4±14.9	not reported	ETN 20.3±7.9<br>PBO 19.4±6.8	BSA≥10% and PGA≥3 and PASI≥12	yes
Reich et.al.,2017	reSURFACE 2<br>(NCT01729754)	132sites<br>(2013.02-2015.09)	12w	50 mg(biw)	313	ETN 45.8±14.0<br>PBO 46.4±12.2	A maximum of 30%	ETN 20.2±7.36<br>PBO 20.0±7.57	BSA≥10% and PGA≥3 and PASI≥12	yes(within 2 months)
**Ustekinumab vs. Placebo**										
Leonardi et.al.,2008	PHOENIX 1<br>(NCT00267969)	48 sites<br>(2005.12-2007.09)	12w	45mg(0w,4w);<br>90mg(0w,4w)	45mg:255;<br>90mg:255	45mg:44.8±12.5;<br>90mg:46.2±11.3;<br>placebo 44.8±11.3	45mg:29.0;<br>90mg: 36.7;<br>placebo 35.3	45mg:20.5±8.6;<br>90mg:19.7±7.6;<br>placebo 20.4±8.6	BSA≥10% and PASI≥12	yes(recent)
Papp et.al.,2008	PHOENIX 2<br>(NCT00307437)	70 sites<br>(2006.03-2007.09)	12w	45mg(0w,4w);<br>90mg(0w,4w)	45mg:409;<br>90mg:411	45mg:45.1±12.1;<br>90mg:46.6±12.1;<br>placebo 47.0±12.5	45mg:26.2;<br>90mg: 22.9;<br>placebo 25.6	45mg:19.4±6.8;<br>90mg: 20.1±7.5;<br>placebo 19.4±7.5	BSA≥10% and PASI≥12	yes(recent)
Tsai et.al.,2011	PEARL	(2008.12-2010.03)	12w	45 mg(0w,4w)	61	UST 40.9±12.7;<br>PBO 40.4±10.1	UST 16.4<br>PBO 11.7	UST 25.2±11.9<br>PBO 22.9±8.6	BSA≥10% and PASI≥12	yes
Igarashi et.al.,2012	/	35 sites<br>(2008.03-2010.03)	12w	45mg(0w,4w);<br>90mg(0w,4w)	45mg:64;<br>90mg:62	45mg:45.0;<br>90mg:44.0;<br>placebo 49.0	45mg:9.4;<br>90mg:11.3;<br>placebo:3.1	45mg:30.1±12.9;<br>90mg: 28.7 ± 11.2;<br>placebo 30.3 ± 11.8	BSA≥10% and PASI≥12	yes
Zhu et.al.,2013	LOTUS	14sites<br>(2009.10-2011.07)	12w	45 mg(0w,4w)	160	UST 40.1±12.4;<br>PBO 39.2 ±12.2	UST 8.8<br>PBO 8.6	UST 23.2±9.5<br>PBO 22.7±9.5	BSA≥10% and PASI≥12	not reported
Lebwohl et.al.,2015	AMAGINE-2<br>(NCT01708603)	142 sites<br>(2012.08-2014.09)	12w	45mg(BW≤100kg) or<br>90mg(BW>100kg)<br>(0w,4w)	300	UST 45.0±13.0;<br>PBO 44.0±13.0	UST 17.0<br>PBO 17.0	UST 20.0±8.4<br>PBO 20.4±8.2	BSA≥10% and sPGA≥3 and PASI≥12	not reported

Lebwohl et.al.,2015	AMAGINE-3 (NCT01708629)	142 sites (2012.09-2014.08)	12w	45mg(BW≤100 kg) or 90mg(BW>100kg) (0w,4w)	313	UST 45.0±13.0; PBO 44.0±13.0	UST 20.0 PBO 19.0	UST 20.1±8.4 PBO 20.1±8.7	BSA≥10% and sPGA≥3 and PASI≥12	not reported
Gordon et.al.,2018	UltIMMA-1 (NCT02684370)	139sites	16w	45 mg(0w,4w)	100	UST 46.5±13.4; PBO 49.3±13.6	UST 23.0 PBO 35.0	UST 20.1±6.8 PBO 20.5±6.7	BSA≥10% and sPGA≥3 and PASI≥12	not reported
Gordon et.al.,2018	UltIMMA-2 (NCT02684357)	139sites	16w	45 mg(0w,4w)	99	UST 48.6±14.8; PBO 46.3±13.3	UST 27.0 PBO 32.0	UST 18.2±5.9 PBO 18.9±7.3	BSA≥10% and sPGA≥3 and PASI≥12	not reported
Reich et.al.,2021	BE VIVID （NCT03370133）	11 countries	16w	45 mg or 90 mg (0w,4w,q12w)	163	UST 46.0±13.6/ PBO 49·7±13.6	not reported	UST 21.3±8.3 PBO 20.1±6.8	PASI score ⩾12, BSA⩾10% , IGA score ⩾3	yes（had a current, or history of, opportunistic, recurrent, or chronic infection; or had active Crohn' s disease or ulcerative colitis）
**Guselkumab vs. Placebo**										
Gordon et.al.,2015	X-PLORE （NCT01483599）	43 sites (2011.10-2013.08)	16w	5mg/50mg/200mg(0w,4w, 12w)/14mg/100mg(q8w)	207	GUS 44.0 PBO 46.5	GUS 25.0 PBO 28.6	GUS 20.0± 8.05 PBO 21.8± 9.98	BSA≥10% and PGA≥3 and PASI≥12	yes(recent)
Blauvelt et.al.,2017	VOYAGE 1 （NCT02207231）	101sites(2014.12-2016.04)	16w	100mg(0w,4w,12w,q8w)	329	GUS 43.9±12.47 PBO 44.9±12.90	GUS 19.5 PBO 17.2	GUS 22.1±9.49 PBO 20.4±8.74	BSA≥10% and IGA≥3 and PASI≥12	yes(recent)
Reich et.al.,2017	VOYAGE 2 (NCT02207244)	115sites(2014.11-2016.05)	16w	100mg(0w,4w,12w,q8w)	494	GUS 43.7±12.2 PBO 43.3±12.4	GUS 17.9 PBO 18.5	GUS 21.9±8.8 PBO 21.5 ±8.0	BSA≥10% and IGA≥3 and PASI≥12	yes
**Biologic agents compared with each other(not mentioned above)**										
Griffiths et.al.,2010	(NCT00454584)	67 sites (2007.03.26-2009.01.15)	12w	ENT 50 mg(biw)<br><br>UST45 or 90mg(0w,4w)	347	ETN45.7±13.4; UST45mg:45.1±12.6; UST90mg:44.8±12.3	ETN 27.4; 45mg:29.7; 90mg:27.4	ETN18.6±6.2; UST45mg:20.5±9.2; UST 90mg:19.9±8.4	BSA≥10% and PGA≥3 and PASI≥12	yes(recent)
Thaci et.al.,2015	CLEAR (NCT02074982)	134 sites (2014.02-2014.10)	16w	SEC 300mg (0w, 1w, 2w, 3w,q4w);<br><br>UST 45mg(BW≤100kg) or 90mg(BW>100kg) (0w,4w)	337	SEC:45.2±13.96; UST:44.6±13.67	SEC:20.5; UST:15.9	SEC:21.7 ±8.5; UST:21.5±8.07	BSA≥10% and IGA≥3 and PASI≥12	yes(within 2 weeks)

Paul et.al.,2019	IXORA-S (NCT02561806)	51 sites (2015.10-2017.05)	52w	IXE 160mg(0w)-80 mg(eow)-80mg(q4w);<br><br>UST 45mg(BW≤100kg) or 90mg(BW>100kg) (0w,4w,16w,28w,40w)	135	IXE:42.7 ±12.7; UST:44.0±13.3	not reported	IXE:19.9 ±8.2; UST:19.8±9.0	PASI score ≥10	not reported
Blauvelt et.al.,2020	IXORA-R (NCT03573323)	multicentre (2018.11-2020.01)	24w	IXE 160 mg(0w)-80 mg(eow,2-12w)-80mg(q4w,14-24w)<br><br>GUS 100 mg (0w,4w,12w,20w)	519	GUS:49.0±14.9/ IXE:49.0±13.9	not reported	GUS:19.3±7.1/ IXE:19.95±7.9	BSA⩾10% and sPGA⩾3 and PASI ⩾12	not reported
Reich et.al.,2019	ECLIPSE (NCT03090100)	142 sites (2017.04-2018.09)	48w	SEC 300mg(0w, 1w, 2w, 3w,q4w)<br><br>GUS 100 mg(0w,4w,q8w)	511	SEC:45.3 ±13.6/ GUS:46.3±13.7	SEC:15/GUS: 18	SEC:20.1±7.6/ GUS:20.0 ±7.4	PASI ≥12, IGA score ≥3, BSA≥10% for ≥6 months	inflammatory bowel disease were excluded

[1] The Criteria of moderate-to-severe psoriasis was defined by the author of each included article.

Figure 1. Forest plot of pooled data of biological agents versus placebo on serious infections. There was no statistically significant difference in the risk of serious infections for patients receiving tumor necrosis factor inhibitors (TNFi), anti-interleukin (IL)-17 agents, or anti-IL-12/23 agents, compared to placebo. (a) TNFi vs. Placebo; (b) Anti-IL-17 agents vs. Placebo; (c) Anti-IL-12/23 agents vs. Placebo.

(a) TNFi vs. Placebo

Study or Subgroup	TNFi Events	TNFi Total	Placebo Events	Placebo Total	Weight	Peto Odds Ratio Peto, Fixed, 95% CI	Year
**5.1.1 Adalimumab vs Placebo**							
Gordon et.al.,2006	1	95	0	52	2.1%	4.70 [0.08, 283.33]	2006
Saurat et.al.,2008	0	107	0	53		Not estimable	2008
Menter et.al.,2008	5	814	4	398	18.0%	0.59 [0.15, 2.38]	2008
Asahina et.al.,2010	1	123	0	46	1.8%	3.95 [0.05, 322.96]	2010
Gordon et.al.,2015	0	43	0	42		Not estimable	2015
Cai et.al.,2017	0	338	0	87		Not estimable	2017
Blauvelt et.al.,2017	2	333	0	174	4.1%	4.60 [0.25, 85.41]	2017
Reich et.al.,2017	2	248	1	248	6.8%	1.95 [0.20, 18.86]	2017
Elewski et.al.,2018	4	109	2	108	13.4%	1.96 [0.39, 9.90]	2018
**Subtotal (95% CI)**		**2210**		**1208**	**46.2%**	**1.41 [0.59, 3.38]**	
Total events	15		7				
Heterogeneity: Chi² = 2.91, df = 5 (P = 0.71); I² = 0%							
Test for overall effect: Z = 0.78 (P = 0.44)							
**5.1.2 Infliximab vs Placebo**							
Gottlieb et.al.,2004	1	197	0	51	1.5%	3.52 [0.03, 449.53]	2004
Reich et.al.,2005	3	298	0	76	4.4%	3.53 [0.21, 59.24]	2005
YANG et.al.,2012	3	84	1	45	8.1%	1.56 [0.19, 12.51]	2012
**Subtotal (95% CI)**		**579**		**172**	**14.0%**	**2.20 [0.45, 10.72]**	
Total events	7		1				
Heterogeneity: Chi² = 0.25, df = 2 (P = 0.88); I² = 0%							
Test for overall effect: Z = 0.98 (P = 0.33)							
**5.1.3 Etanercept vs Placebo**							
Gottlieb et.al.,2003	1	57	1	55	4.5%	0.96 [0.06, 15.62]	2003
Papp et.al.,2005	0	390	1	193	2.0%	0.05 [0.00, 3.14]	2005
Tyring et.al.,2006	0	312	1	306	2.3%	0.13 [0.00, 6.69]	2006
van de Kerkhof et.al.,2008	0	96	0	46		Not estimable	2008
Strober et.al.,2011	0	139	0	72		Not estimable	2011
Gottlieb et.al.,2011	1	141	0	68	2.0%	4.40 [0.07, 288.78]	2011
Langley et.al.,2014 FIXTURE (ETN)	4	323	1	327	11.3%	3.39 [0.58, 19.66]	2014
Griffiths et.al.,2015 UNCOVER-2	3	358	0	168	5.9%	4.37 [0.38, 49.73]	2015
Bachelez et.al.,2015	2	335	0	107	3.3%	3.75 [0.15, 95.73]	2015
Griffiths et.al.,2015 UNCOVER-3	0	382	2	193	4.1%	0.05 [0.00, 0.95]	2015
Reich et.al.,2017 reSURFACE 2	0	313	1	156	2.0%	0.05 [0.00, 3.17]	2017
Reich et.al.,2017 LIBERATE	1	83	0	84	2.3%	7.48 [0.15, 376.93]	2017
**Subtotal (95% CI)**		**2929**		**1775**	**39.8%**	**1.15 [0.45, 2.93]**	
Total events	12		7				
Heterogeneity: Chi² = 14.33, df = 9 (P = 0.11); I² = 37%							
Test for overall effect: Z = 0.29 (P = 0.77)							
**Total (95% CI)**		**5718**		**3155**	**100.0%**	**1.38 [0.77, 2.50]**	
Total events	34		15				
Heterogeneity: Chi² = 17.98, df = 18 (P = 0.46); I² = 0%							
Test for overall effect: Z = 1.08 (P = 0.28)							
Test for subgroup differences: Chi² = 0.49, df = 2 (P = 0.78), I² = 0%							

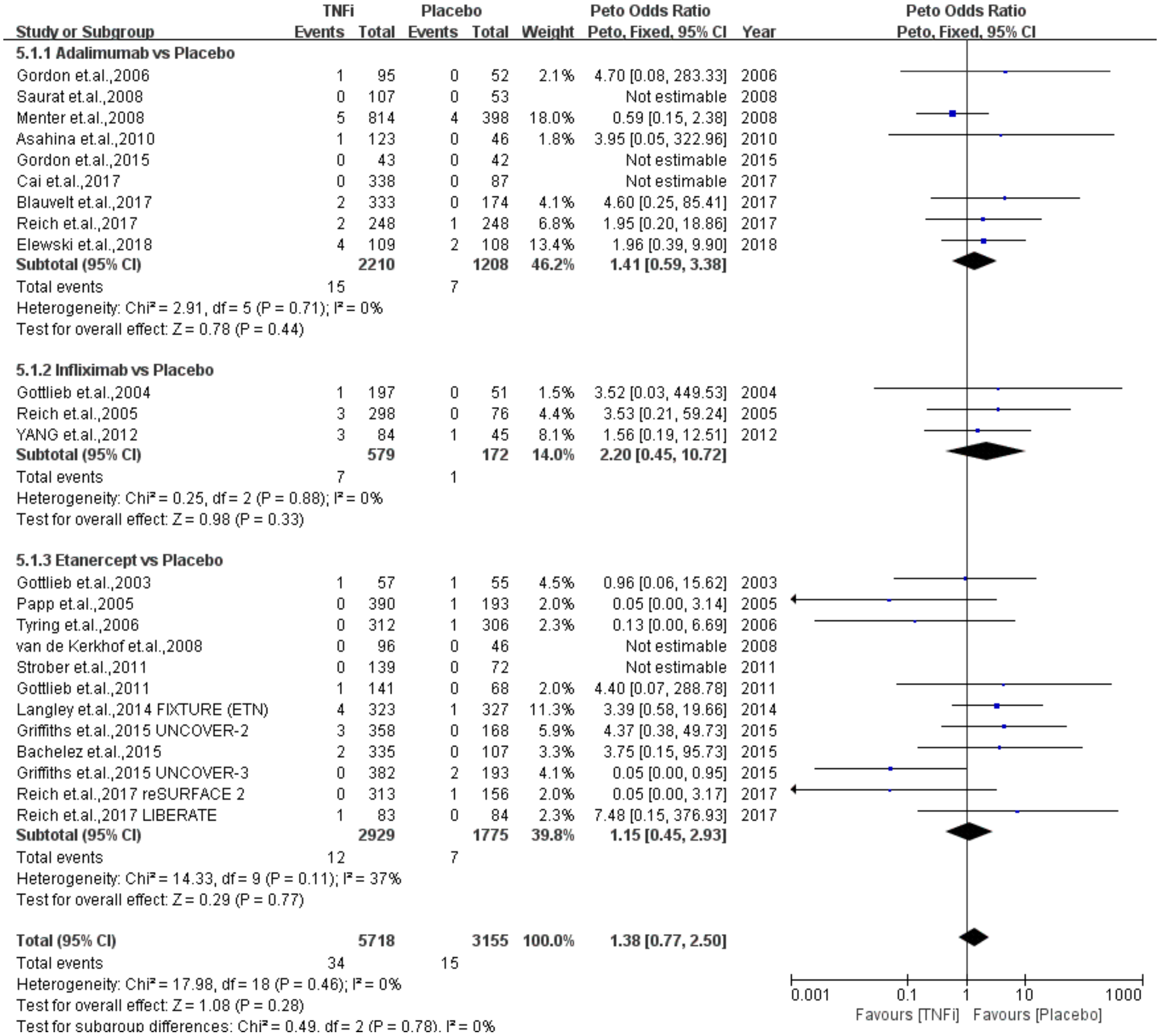

(b) Anti-IL-17 agents vs. Placebo

Study or Subgroup	Anti-IL-17 agents Events	Anti-IL-17 agents Total	Placebo Events	Placebo Total	Weight	Peto Odds Ratio Peto, Fixed, 95% CI	Year
**6.1.1 Secukinumab vs Placebo**							
Rich et.al.,2013	2	337	0	67	3.2%	3.33 [0.08, 138.74]	2013
Langley et.al.,2014 ERASURE (SEC)	0	490	1	247	2.6%	0.05 [0.00, 3.22]	2014
Langley et.al.,2014 FIXTURE (SEC)	1	654	1	327	5.2%	0.47 [0.02, 8.94]	2014
Paul et.al.,2015	1	121	0	61	2.6%	4.50 [0.07, 286.05]	2015
Gottlieb et.al.,2016	2	118	1	59	7.8%	1.00 [0.09, 11.18]	2016
Bagel et.al.,2017	0	51	1	51	2.9%	0.14 [0.00, 6.82]	2017
Stebut et.al.,2019	1	102	0	49	2.6%	4.39 [0.07, 289.07]	2019
**Subtotal (95% CI)**		**1873**		**861**	**27.0%**	**0.80 [0.22, 2.92]**	
Total events	7		4				
Heterogeneity: Chi² = 4.51, df = 6 (P = 0.61); I² = 0%							
Test for overall effect: Z = 0.33 (P = 0.74)							
**6.1.2 Ixekizumab vs Placebo**							
Leonardi et.al.,2012	0	115	0	27		Not estimable	2012
Gordon et.al.,2016	13	2328	3	791	35.5%	1.42 [0.46, 4.39]	2016
Ryan et.al.,2018	1	75	0	74	2.9%	7.29 [0.14, 367.49]	2018
**Subtotal (95% CI)**		**2518**		**892**	**38.4%**	**1.61 [0.54, 4.76]**	
Total events	14		3				
Heterogeneity: Chi² = 0.62, df = 1 (P = 0.43); I² = 0%							
Test for overall effect: Z = 0.86 (P = 0.39)							
**6.1.3 Brodalumab vs Placebo**							
Papp et.al.,2012	0	160	0	38		Not estimable	2012
Lebwohl et.al.,2015 AMAGINE-2 (BRO)	4	1222	1	309	9.5%	1.01 [0.11, 9.01]	2015
Lebwohl et.al.,2015 AMAGINE-3 (BRO)	7	1253	1	315	15.1%	1.61 [0.28, 9.10]	2015
Papp et.al.,2016	3	441	0	220	7.8%	4.50 [0.41, 49.82]	2016
Nakagawa et.al.,2016	1	113	0	38	2.2%	3.80 [0.04, 348.17]	2016
**Subtotal (95% CI)**		**3189**		**920**	**34.6%**	**1.89 [0.60, 5.93]**	
Total events	15		2				
Heterogeneity: Chi² = 0.94, df = 3 (P = 0.82); I² = 0%							
Test for overall effect: Z = 1.09 (P = 0.28)							
**Total (95% CI)**		**7580**		**2673**	**100.0%**	**1.41 [0.72, 2.76]**	
Total events	36		9				
Heterogeneity: Chi² = 7.10, df = 12 (P = 0.85); I² = 0%							
Test for overall effect: Z = 1.00 (P = 0.32)							
Test for subgroup differences: Chi² = 1.04, df = 2 (P = 0.59), I² = 0%							

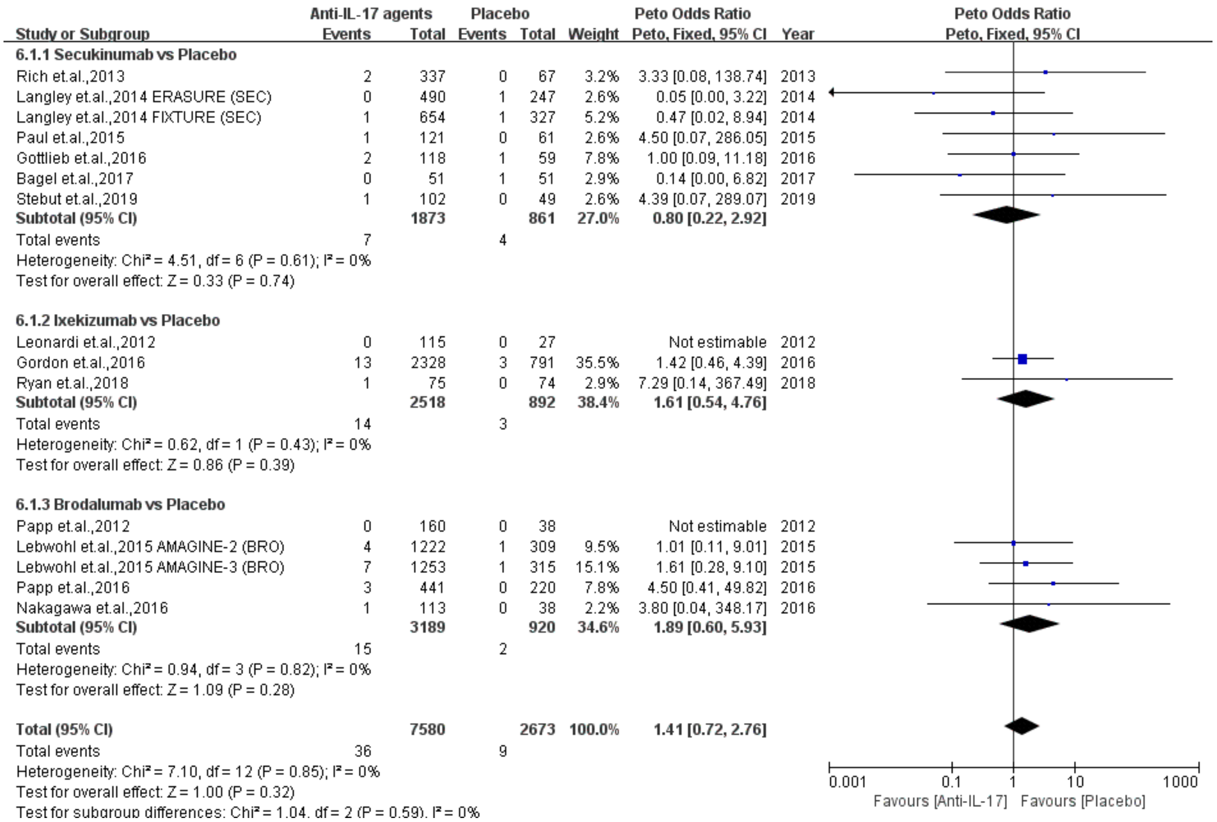

(c) Anti-IL-12/23 agents vs. Placebo

Study or Subgroup	Favours [Anti-IL-12/23] Events	Favours [Anti-IL-12/23] Total	Placebo Events	Placebo Total	Weight	Peto Odds Ratio Peto, Fixed, 95% CI
**7.1.1 Ustekinumab vs Placebo**						
Gordon et.al.,2018 UltIMMA-1	3	100	0	102	15.2%	7.69 [0.79, 74.81]
Gordon et.al.,2018 UltIMMA-2	1	99	0	98	5.1%	7.31 [0.15, 368.66]
Igarashi et.al.,2012	1	126	0	32	3.3%	3.50 [0.03, 459.84]
Lebwohl et.al.,2015 AMAGINE-2 (UST)	0	300	1	309	5.1%	0.14 [0.00, 7.03]
Lebwohl et.al.,2015 AMAGINE-3 (UST)	2	313	1	315	15.3%	1.96 [0.20, 18.95]
Leonardi et.al.,2008	2	510	1	255	13.6%	1.00 [0.09, 11.06]
Papp et.al.,2008	1	820	2	410	13.6%	0.22 [0.02, 2.46]
Reich et.al.,2021	2	163	0	83	9.1%	4.55 [0.24, 85.84]
Tsai et.al.,2011	0	61	1	60	5.1%	0.13 [0.00, 6.71]
Zhu et.al.,2013	0	160	0	161		Not estimable
**Subtotal (95% CI)**		**2652**		**1825**	**85.2%**	**1.40 [0.54, 3.65]**
Total events	12		6			
Heterogeneity: Chi² = 8.72, df = 8 (P = 0.37); I² = 8%						
Test for overall effect: Z = 0.68 (P = 0.49)						
**7.1.2 Guselkumab vs Placebo**						
Blauvelt et.al.,2017(GUS)	0	329	0	174		Not estimable
Gordon et.al.,2015(GUS)	2	208	0	42	5.7%	3.34 [0.08, 137.17]
Reich et.al.,2017(GUS)	1	494	1	248	9.1%	0.47 [0.03, 8.97]
**Subtotal (95% CI)**		**1031**		**464**	**14.8%**	**1.01 [0.10, 10.09]**
Total events	3		1			
Heterogeneity: Chi² = 0.65, df = 1 (P = 0.42); I² = 0%						
Test for overall effect: Z = 0.01 (P = 1.00)						
**Total (95% CI)**		**3683**		**2289**	**100.0%**	**1.33 [0.55, 3.23]**
Total events	15		7			
Heterogeneity: Chi² = 9.44, df = 10 (P = 0.49); I² = 0%						
Test for overall effect: Z = 0.63 (P = 0.53)						
Test for subgroup differences: Chi² = 0.07, df = 1 (P = 0.80), I² = 0%						

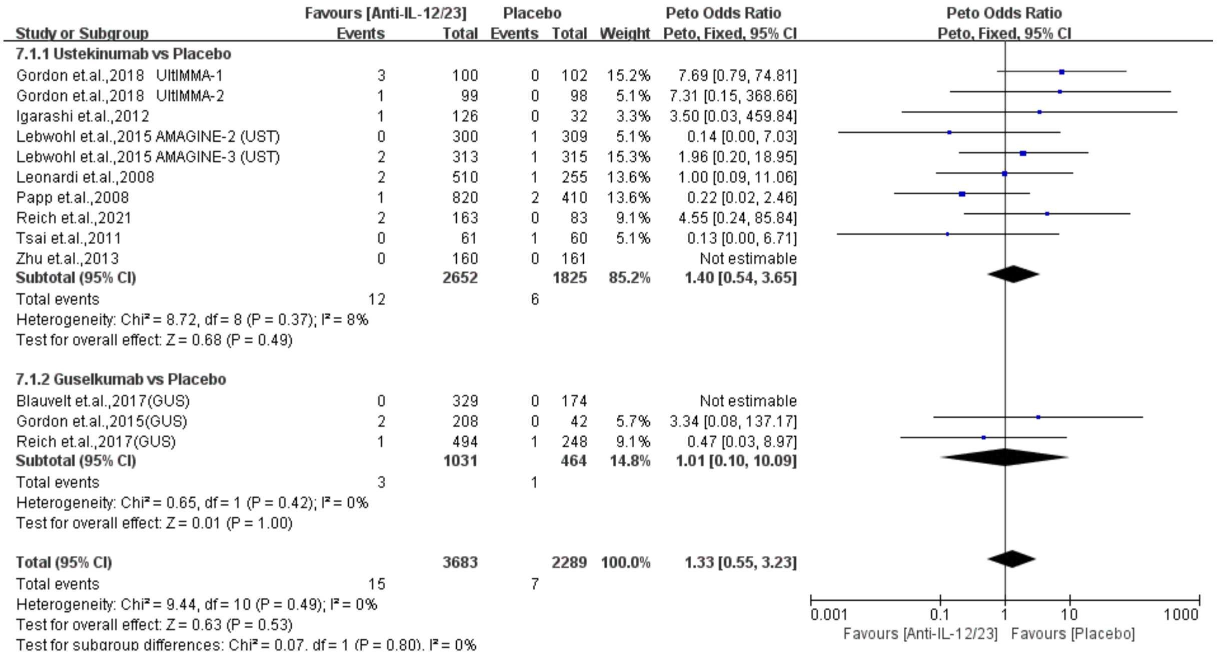

Figure 2. Forest plot of pooled data of biologic agents comparing to each other on serious infections. No significant difference in the risk of serious infection among different biologic agents was detected. (a) Anti-interleukin (IL)-17 agents vs. tumor necrosis factor inhibitors (TNFi); (b) Anti-IL-17 agents vs. Anti-IL-12/23 agents; (c) Anti-IL-12/23 agents vs. TNFi.

(a) Anti-IL-17 agents vs. TNFi

	Anti-IL-17 agents		TNFi			Peto Odds Ratio
Study or Subgroup	Events	Total	Events	Total	Weight	Peto, Fixed, 95% CI
Griffiths et.al.,2015	8	936	4	323	43.8%	0.67 [0.18, 2.45]
Langley et.al.,2014 FIXTURE	13	2328	3	740	56.2%	1.34 [0.43, 4.23]
**Total (95% CI)**		**3264**		**1063**	**100.0%**	**0.99 [0.42, 2.34]**
Total events	21		7			
Heterogeneity: Chi² = 0.63, df = 1 (P = 0.43); I² = 0%						
Test for overall effect: Z = 0.03 (P = 0.98)						

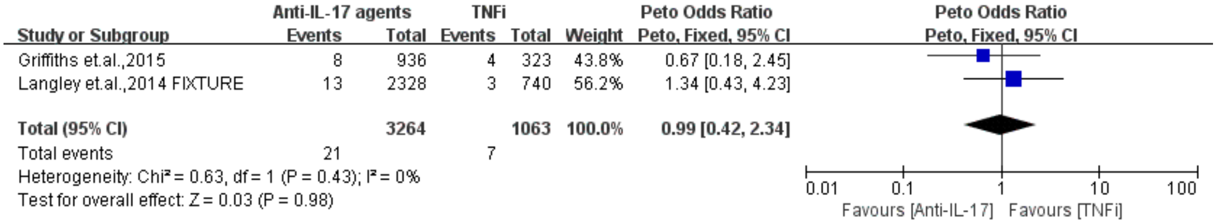

(b) Anti-IL-17 agents vs. Anti-IL-12/23 agents

	Anti-IL-17 agents		Anti-IL-12/23 agents			Peto Odds Ratio
Study or Subgroup	Events	Total	Events	Total	Weight	Peto, Fixed, 95% CI
Blauvelt et.al.,2020	2	519	2	506	15.3%	0.97 [0.14, 6.94]
Lebwohl et.al.,2015 AMAGINE-2 (BRO)	4	1222	0	300	9.7%	3.48 [0.30, 41.01]
Lebwohl et.al.,2015 AMAGINE-3 (BRO)	7	1253	2	313	21.9%	0.87 [0.17, 4.47]
Reich et.al.,2019	5	511	6	534	41.7%	0.87 [0.27, 2.85]
Thaci et.al.,2015	1	337	2	339	11.4%	0.52 [0.05, 4.97]
**Total (95% CI)**		**3842**		**1992**	**100.0%**	**0.95 [0.44, 2.05]**
Total events	19		12			
Heterogeneity: Chi² = 1.38, df = 4 (P = 0.85); I² = 0%						
Test for overall effect: Z = 0.12 (P = 0.90)						

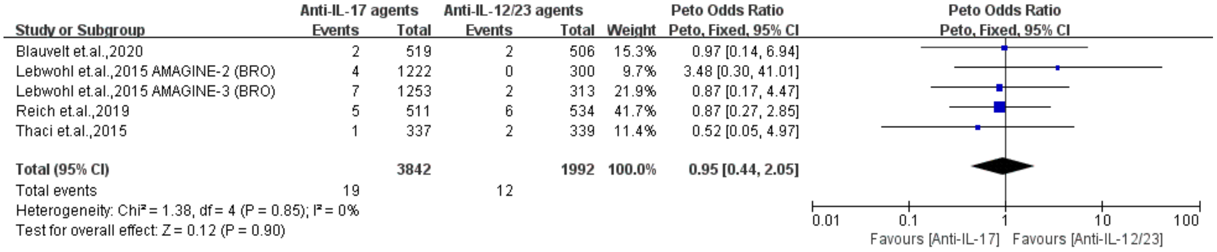

(c) Anti-IL-12/23 agents vs. TNFi

	Anti-IL-12/23 agents		TNFi			Peto Odds Ratio
Study or Subgroup	Events	Total	Events	Total	Weight	Peto, Fixed, 95% CI
Blauvelt et.al.,2017(GUS)	3	333	2	329	30.8%	1.48 [0.25, 8.57]
Gordon et.al.,2015(GUS)	0	43	2	207	7.0%	0.30 [0.01, 11.79]
Griffiths et.al.,2010	4	556	1	347	29.2%	2.19 [0.36, 13.31]
Reich et.al.,2017(GUS)	3	248	3	494	32.9%	2.12 [0.39, 11.61]
**Total (95% CI)**		**1180**		**1377**	**100.0%**	**1.67 [0.63, 4.42]**
Total events	10		8			
Heterogeneity: Chi² = 1.02, df = 3 (P = 0.80); I² = 0%						
Test for overall effect: Z = 1.02 (P = 0.31)						

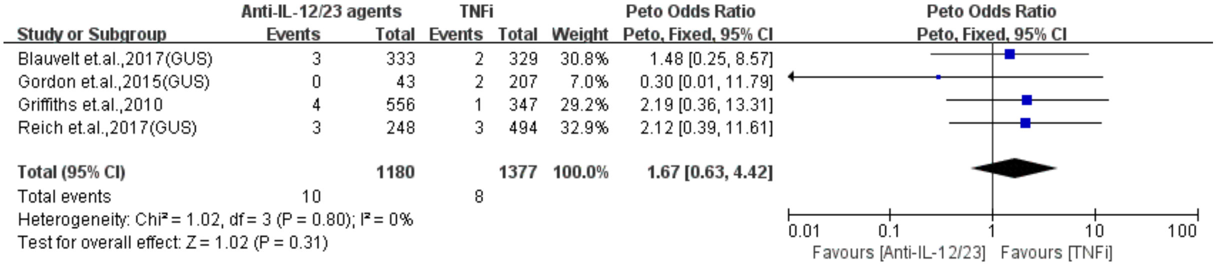

Table 3. Risk of bias assessment for randomize controlled trials (RCTs).

Authors, year	Sequence generation	Allocation concealment	Blinding of participants and personnel	Blinding of outcome assessors	Incomplete outcome data	Selective outcome reporting	Other bias
**Anti-IL-17 agents vs. Placebo - Candida infections**							
**Secukinumab vs. placebo**							
Langley et.al., 2014 (ERASURE)	Low	Low	Low	Low	Low	Low	Low
Langley et.al., 2014 (FIXTURE)	Low	Low	Low	Low	Low	Low	Low
Paul et.al., 2015 (JUNCTURE)	Low	Low	Low	Low	Low	Low	Low
Gottlieb et.al.,2016	Low	Low	Low	Low	Low	Low	Low
Bagel et.al.,2017	Low	Low	Low	Low	Low	Low	Low
von Stebut et.al.,2019	Low	Low	Low	Low	Low	Low	Low
**Ixekizumab vs. placebo**							
Gordon et.al., 2016 (UNCOVER)	Low	Low	Low	Low	Low	Low	Low
**Brodalumab vs. placebo**							
Lebwohl et.al.a., 2015 (AMAGINE 2)	Low	Low	Low	Low	Low	Low	Low
Lebwohl et.al.b., 2015 (AMAGINE 3)	Low	Low	Low	Low	Low	Low	Low
Nakagawa et.al.,2016	Low	Low	Low	High	High	Low	Unclear
Papp et.al.,2016	Low	Low	Low	Low	Low	Low	Low
**Biologic agents vs. Biologic agents - Candida infections**							
Langley et.al., 2014 (FIXTURE)	Low	Low	Low	Low	Low	Low	Low
Lebwohl et.al.a., 2015 (AMAGINE 2)	Low	Low	Low	Low	Low	Low	Low
Lebwohl et.al.b., 2015 (AMAGINE 3)	Low	Low	Low	Low	Low	Low	Low
Griffiths et.al.,2015	Low	Low	Low	Low	Low	Low	Low
Thaci et.al.,2015	Low	Low	Low	Low	Low	Low	Low
Paul et.al.,2019	Low	Low	Low	High	Low	Low	Unclear
Reich et.al.,2019	Low	Low	Low	Low	Low	Low	Low
Blauvelt et.al.,2020	Low	Low	Low	Low	Low	Low	Low
**Biologic agents vs. Placebo - Serious infections**							
**Adalimumab vs. placebo**							
Gordon et.al.,2006	Low	Low	Low	High	Low	Low	Low
Menter et.al., 2008 (REVEAL)	Low	Low	Unclear	Low	Low	Unclear	Unclear
Saurat et.al., 2008 (CHAMPION)	Low	Low	Low	Low	Low	Unclear	Low
Asahina et.al.,2010	Low	Low	Low	Unclear	Low	Low	Low
Gordon et.al., 2015 (X-PLORE)	Unclear	Unclear	High	Unclear	Low	Unclear	Unclear
Cai et.al.,2017	Unclear	Unclear	Unclear	Unclear	Low	Unclear	Unclear
Blauvelt et.al.,2017	Low	Low	Low	Low	Low	Low	Low
Reich et.al.,2017	Low	Low	Low	Low	Low	Low	Low
Elewski et.al.,2018	Low	Low	Low	Unclear	Low	Low	Low
**Infliximab vs. placebo**							
Gottlieb et.al., 2004 (SPIRIT)	Low	Low	Low	Unclear	High	Unclear	Unclear
Reich et.al., 2005 (EXPRESS)	Low	Low	Low	Low	Low	Unclear	Unclear
Yang et.al., 2012	Unclear	Unclear	Unclear	Unclear	Low	Unclear	Unclear

Authors, year	Sequence generation	Allocation concealment	Blinding of participants and personnel	Blinding of outcome assessors	Incomplete outcome data	Selective outcome reporting	Other bias
**Etanercept vs. placebo**							
Gottlieb et.at., 2003	Low	Low	Low	Low	High	Unclear	Unclear
Papp et.al.,2005	Low	Low	Low	Low	Low	Unclear	Unclear
Tyring et.al., 2006	Low	Low	Unclear	Unclear	Low	Low	Unclear
van de Kerkhof et.al., 2008	Low	Low	Unclear	Unclear	Low	Unclear	Unclear
Gottlieb et.al., 2011	Unclear	Unclear	Unclear	Unclear	Low	Low	Unclear
Strober et.al., 2011	Unclear	Unclear	Unclear	Unclear	Low	Low	Unclear
Langley et.al., 2014 (FIXTURE)	Low	Low	Low	Low	Low	Low	Low
Bachelez et.al., 2015	Low	Low	Low	Low	Low	Low	Low
Griffiths et.al.,2015(UNCOVER-2)	Low	Low	Low	Unclear	Low	Low	Low
Griffiths et.al.,2015(UNCOVER-3)	Low	Low	Low	Low	Low	Low	Low
Reich et.al.,2017 (reSurface2)	Low	Low	Low	Low	Low	Low	Low
Reich et.al.,2017 (LIBERATE)	Low	Low	Low	Low	Low	Low	Low
**Ustekinumab vs. placebo**							
Leonardi et.al., 2008 (PHOENIX 1)	Low	Low	Low	Low	Low	Unclear	Unclear
Papp et.al., 2008 (PHOENIX 2)	Low	Low	Low	Unclear	Low	Unclear	Unclear
Tsai et.al., 2011 (PEARL)	Low	Low	Low	Unclear	Low	Unclear	Unclear
Igarashi et.al, 2012	Unclear	Unclear	Unclear	Unclear	Low	Unclear	Unclear
Zhu et.al., 2013 (LOTUS)	Unclear	Unclear	Unclear	Unclear	Low	Low	Unclear
Lebwohl et.al.a., 2015 (AMAGINE 2)	Low	Low	Low	Low	Low	Low	Low
Lebwohl et.al.b., 2015 (AMAGINE 3)	Low	Low	Low	Low	Low	Low	Low
Gordon et.al.,2018 (UltIMMA-1)	Low	Low	Low	Low	Low	Low	Low
Gordon et.al.,2018 (UltIMMA-2)	Low	Low	Low	Unclear	Low	Low	Low
Reich et.al.,2021	Low	Low	Low	Low	Low	Low	Low
**Guselkumab vs. placebo**							
Gordon et.al.,2015	Unclear	Unclear	High	Unclear	Low	Unclear	Unclear
Blauvelt et.al.,2017	Low	Low	Low	Low	Low	Low	Low
Reich et.al.,2017	Low	Low	Low	Low	Low	Low	Low
**Secukinumab vs. placebo**							
Rich et.al.,2013	Low	Low	Low	Unclear	Low	Low	Low
Langley et.al., 2014 (ERASURE)	Low	Low	Low	Low	Low	Low	Low
Langley et.al., 2014 (FIXTURE)	Low	Low	Low	Low	Low	Low	Low
Paul et.al., 2015 (JUNCTURE)	Low	Low	Low	Low	Low	Low	Unclear
Gottlieb et.al.,2016 (FEATURE)	Low	Low	Low	Low	Low	Low	Low
Bagel et.al.,2017	Low	Low	Low	Low	Low	Low	Unclear
von Stebut et.al.,2019	Low	Low	Low	Low	Low	Low	Low
**Ixekizumab vs. placebo**							
Leonardi et.al.,2012	Low	Low	Low	Low	Low	Low	Low
Gordon et.al., 2016 (UNCOVER)	Low	Low	Low	Low	Low	Low	Low
Ryan et.al.,2018	Low	Low	Low	Unclear	Low	Low	Low
**Brodalumab vs. placebo**							

Authors, year	Sequence generation	Allocation concealment	Blinding of participants and personnel	Blinding of outcome assessors	Incomplete outcome data	Selective outcome reporting	Other bias
Papp et.al.,2012	Low	Low	Low	Low	Low	Low	Low
Lebwohl et.al.a., 2015 (AMAGINE 2)	Low	Low	Low	Low	Low	Low	Low
Lebwohl et.al.b., 2015 (AMAGINE 3)	Low	Low	Low	Low	Low	Low	Low
Nakagawa et.al.,2016	Low	Low	Low	High	High	Low	Unclear
Papp et.al.,2016	Low	Low	Low	Low	Low	Low	Low
**Biologic agents vs. Biologic agents - Serious infections**							
Griffiths et.al., 2010	Low	Low	Unclear	Low	Low	Low	Low
Langley et.al., 2014 (FIXTURE)	Low	Low	Low	Low	Low	Low	Low
Gordon et.al.,2015	Unclear	Unclear	High	Unclear	Low	Unclear	Unclear
Lebwohl et.al.a., 2015 (AMAGINE 2)	Low	Low	Low	Low	Low	Low	Low
Lebwohl et.al.b., 2015 (AMAGINE 3)	Low	Low	Low	Low	Low	Low	Low
Thaci et.al.,2015	Low	Low	Low	Low	Low	Low	Low
Griffiths et.al., 2015(UNCOVER)	Low	Low	Low	Low	Low	Low	Low
Blauvelt et.al.,2017	Low	Low	Low	Low	Low	Low	Low
Reich et.al.,2017	Low	Low	Low	Low	Low	Low	Low
Paul et.al.,2019	Low	Low	Low	Low	Low	Low	Low
Reich et.al.,2019	Low	Low	Low	Low	Low	Low	Low
Blauvelt et.al.,2020	Low	Low	Low	Low	Low	Low	Low
**Biologic agents vs. Methotrexate - Serious infections**							
Saurat et.al.,2008	Low	Low	Low	Low	Low	Unclear	Low
Reich et.al.,2019	Low	High	High	Low	Low	Low	Low

Table 4. Quality of evidence on *Candid*a infections using Grades of Recommendations Assessment, Development and Evaluation (GRADE). (a) Anti-interleukin (IL)-17 agents vs. Placebo; (b) Anti-IL-17 agents vs. Anti-IL-12/23 agents; (c) Anti-IL-17 agents vs. tumor necrosis factor inhibitors (TNFi).

(a) Anti-IL-17 agents vs. Placebo

**Anti-IL-17 agents vs. Placebo - Candida infections**											
**Quality assessment**							**Summary of Findings**				
**Participants (studies) Follow up**	**Risk of bias**	**Inconsistency**	**Indirectness**	**Imprecision**	**Publication bias**	**Overall quality of evidence**	**Study event rates (%)**		**Relative effect** (95% CI)	**Anticipated absolute effects**	
							**With Placebo-Candida infection**	**With Anti-IL-17 agents**		**Risk with Placebo - Candida infection**	**Risk difference with Anti-IL-17 agents** (95% CI)
**Anti-IL-17 agents vs. Placebo for candida infection**											
9852 (11 studies)	no serious risk of bias	no serious inconsistency	no serious indirectness	no serious imprecision	undetected	⊕⊕⊕⊕ **HIGH**	13/2465 (0.5%)	113/7387 (1.5%)	**OR 2.3** (1.54 to 3.45)	**Study population**	
										**5 per 1000**	**7 more per 1000** (from 3 more to 13 more)
										**Moderate**	
										**4 per 1000**	**5 more per 1000** (from 2 more to 10 more)
**Anti-IL-17 agents vs. Placebo for candida infection - Secukinumab vs. Placebo**											
2824 (6 studies)	no serious risk of bias	no serious inconsistency	no serious indirectness	no serious imprecision	undetected	⊕⊕⊕⊕ **HIGH**	3/794 (0.4%)	54/2030 (2.7%)	**OR 3.22** (1.79 to 5.8)	**Study population**	
										**4 per 1000**	**8 more per 1000** (from 3 more to 18 more)
										**Moderate**	
										**2 per 1000**	**4 more per 1000** (from 2 more to 9 more)

**Anti-IL-17 agents vs. Placebo for candida infection - Ixekizumab vs. Placebo**											
3119<br>(1 study)	no serious risk of bias	no serious inconsistency	no serious indirectness	very serious[1]	undetected	⊕⊕⊝⊝<br>**LOW**[1]<br>due to imprecision	4/791<br>(0.5%)	23/2328<br>(1%)	**OR 1.75**<br>(0.73 to 4.19)	**Study population**	
										**5 per 1000**	**4 more per 1000**<br>(from 1 fewer to 16 more)
										**Moderate**	
										**5 per 1000**	**4 more per 1000**<br>(from 1 fewer to 16 more)
**Anti-IL-17 agents vs. Placebo for candida infection - Brodalumab vs. Placebo**											
3909<br>(4 studies)	no serious risk of bias	no serious inconsistency	no serious indirectness	very serious[1]	undetected	⊕⊕⊝⊝<br>**LOW**[1]<br>due to imprecision	6/880<br>(0.7%)	36/3029<br>(1.2%)	**OR 1.66**<br>(0.8 to 3.44)	**Study population**	
										**7 per 1000**	**4 more per 1000**<br>(from 1 fewer to 16 more)
										**Moderate**	
										**5 per 1000**	**3 more per 1000**<br>(from 1 fewer to 12 more)

[1] Very serious imprecision-lower boundary indicates appreciable benefit while upper boundary indicates appreciable harm.

(b) Anti-IL-17 agents vs. Anti-IL-12/23 agents

Anti-IL-17 agents vs. Anti-IL-12/23 agents - Candida infection											
**Quality assessment**							**Summary of Findings**				
**Participants (studies) Follow up**	**Risk of bias**	**Inconsistency**	**Indirectness**	**Imprecision**	**Publication bias**	**Overall quality of evidence**	**Study event rates (%)**		**Relative effect** (95% CI)	**Anticipated absolute effects**	
							**With Anti-IL-12/23 agents for Candida infection**	**With Anti-IL-17 agents**		**Risk with Anti-IL-12/23 agents for Candida infection**	**Risk difference with Anti-IL-17 agents** (95% CI)
**Anti-IL-17 agents vs. Anti-IL-12/23 agents**											
6130 (6 studies)	no serious risk of bias	no serious inconsistency	no serious indirectness	serious[1]	undetected	⊕⊕⊕⊝ **MODERATE**[1] due to imprecision	23/2155 (1.1%)	84/3975 (2.1%)	**OR 2.55** (1.69 to 3.83)	**Study population**	
										**11 per 1000**	**16 more per 1000** (from 7 more to 29 more)
										**Moderate**	
										**11 per 1000**	**17 more per 1000** (from 7 more to 30 more)

[1] Serious imprecision-the 95% CI is wide, however, without containing 1.

(c) Anti-IL-17 agents vs. TNFi

Anti-IL-17 agents vs. TNFi - Candida infection											
**Quality assessment**							**Summary of Findings**				
**Participants (studies) Follow up**	**Risk of bias**	**Inconsistency**	**Indirectness**	**Imprecision**	**Publication bias**	**Overall quality of evidence**	**Study event rates (%)**		**Relative effect** (95% CI)	**Anticipated absolute effects**	
							**With TNFi - Candida infection**	**With Anti-IL-17 agents**		**Risk with TNFi - Candida infection**	**Risk difference with Anti-IL-17 agents** (95% CI)
**Anti-IL-17 agents vs. TNFi**											
4327 (2 studies)	no serious risk of bias	no serious inconsistency	no serious indirectness	very serious[1]	undetected	⊕⊕⊝⊝ **LOW**[1] due to imprecision	9;1063 (0.8%)	49;3264 (1.5%)	**OR 1.68** (0.92 to 3.07)	**Study population**	
										**8 per 1000**	**6 more per 1000** (from 1 fewer to 17 more)
										**Moderate**	
										**10 per 1000**	**7 more per 1000** (from 1 fewer to 20 more)

[1] Very serious imprecision-lower boundary indicates appreciable benefit while upper boundary indicates appreciable harm.

Table 5. Quality of evidence on serious infections using Grades of Recommendations Assessment, Development and Evaluation (GRADE). (a) Biologics vs. Placebo; (b) tumor necrosis factor inhibitors (TNFi) vs. Placebo; (c) Anti-interleukin (IL)-17 agents vs. Placebo; (d) Anti-IL-12/23 agents vs. Placebo; (e) Anti-IL-17 agents vs. TNFi; (f) Anti-IL-17 agents vs. Anti-IL-12/23 agents; (g) Anti-IL-12/23 agents vs. TNFi.

(a) Biologics vs. Placebo

**Biologics vs. Placebo - Serious infections**											
**Quality assessment**							**Summary of Findings**				
**Participants (studies) Follow up**	**Risk of bias**	**Inconsistency**	**Indirectness**	**Imprecision**	**Publication bias**	**Overall quality of evidence**	**Study event rates (%)**		**Relative effect** (95% CI)	**Anticipated absolute effects**	
							**With Placebo**	**With Biologics**		**Risk with Placebo**	**Risk difference with Biologics** (95% CI)
**Biologics vs Placebo**											
25098 (52 studies)	serious[1]	no serious inconsistency	no serious indirectness	very serious[2]	undetected	⊕⊝⊝⊝ **VERY LOW**[1,2] due to risk of bias, imprecision	31/8117 (0.4%)	85/16981 (0.5%)	**OR 1.38** (0.93 to 2.06)	**Study population**	
										**4 per 1000**	**1 more per 1000** (from 0 fewer to 4 more)
										**Moderate**	
										**0 per 1000**	-

[1] Information from studies at high risk of bias.
[2] Very serious imprecision-lower boundary indicates appreciable benefit while upper boundary indicates appreciable harm.

(b) TNFi vs. Placebo

TNFi vs. placebo - Serious infection											
**Quality assessment**							**Summary of Findings**				
**Participants (studies) Follow up**	**Risk of bias**	**Inconsistency**	**Indirectness**	**Imprecision**	**Publication bias**	**Overall quality of evidence**	**Study event rates (%)**		**Relative effect** (95% CI)	**Anticipated absolute effects**	
							**With Placebo**	**With TNFi**		**Risk with Placebo**	**Risk difference with TNFi** (95% CI)
**TNFi vs. Placebo**											
8873 (24 studies)	serious[1]	no serious inconsistency	no serious indirectness	very serious[2]	undetected	⊕⊖⊖⊖ **VERY LOW**[1,2] due to risk of bias, imprecision	15;3155 (0.5%)	34;5718 (0.6%)	**OR 1.38** (0.77 to 2.5)	**Study population**	
										**5 per 1000**	**2 more per 1000** (from 1 fewer to 7 more)
										**Moderate**	
										**0 per 1000**	-
**TNFi vs. Placebo - Adalimumab vs. Placebo**											
3418 (9 studies)	serious[1]	no serious inconsistency	no serious indirectness	very serious[2]	undetected	⊕⊖⊖⊖ **VERY LOW**[1,2] due to risk of bias, imprecision	7;1208 (0.6%)	15;2210 (0.7%)	**OR 1.41** (0.59 to 3.38)	**Study population**	
										**6 per 1000**	**2 more per 1000** (from 2 fewer to 14 more)
										**Moderate**	
										**0 per 1000**	-
**TNFi vs. Placebo - Infliximab vs. Placebo**											
751 (3 studies)	serious[1]	no serious inconsistency	no serious indirectness	very serious[2]	undetected	⊕⊖⊖⊖ **VERY LOW**[1,2] due to risk of bias, imprecision	1;172 (0.6%)	7;579 (1.2%)	**OR 2.2** (0.45 to 10.72)	**Study population**	
										**6 per 1000**	**7 more per 1000** (from 3 fewer to 53 more)
										**Moderate**	
										**0 per 1000**	-

**TNFi vs. Placebo - Etanercept vs. Placebo**											
4704 (12 studies)	no serious risk of bias	no serious inconsistency	no serious indirectness	very serious[2]	undetected	⊕⊕⊝⊝ **LOW**[2] due to imprecision	7;1775 (0.4%)	12;2929 (0.4%)	**OR 1.15** (0.45 to 2.93)	**Study population**	
										**4 per 1000**	**1 more per 1000** (from 2 fewer to 8 more)
										**Moderate**	
										**2 per 1000**	**0 more per 1000** (from 1 fewer to 4 more)

[1] Information from studies at high risk of bias.

[2] Very serious imprecision-lower boundary indicates appreciable benefit while upper boundary indicates appreciable harm.

(c) Anti-IL-17 agents vs. Placebo

Anti-IL-17 agents vs. Placebo - Serious infection											
**Quality assessment**							**Summary of Findings**				
**Participants (studies) Follow up**	**Risk of bias**	**Inconsistency**	**Indirectness**	**Imprecision**	**Publication bias**	**Overall quality of evidence**	**Study event rates (%)**		**Relative effect** (95% CI)	**Anticipated absolute effects**	
							**With Placebo**	**With Anti-IL-17 agents**		**Risk with Placebo**	**Risk difference with Anti-IL-17 agents** (95% CI)
**Anti-IL-17 agents subgroup vs. Placebo**											
10253 (15 studies)	no serious risk of bias	no serious inconsistency	no serious indirectness	very serious[1]	undetected	⊕⊕⊝⊝ **LOW**[1] due to imprecision	9/2673 (0.3%)	36/7580 (0.5%)	**OR 1.41** (0.72 to 2.76)	**Study population**	
										**3 per 1000**	**1 more per 1000** (from 1 fewer to 6 more)
										**Moderate**	
										**0 per 1000**	-
**Anti-IL-17 agents subgroup vs. Placebo - Secukinumab vs. Placebo**											
2734 (7 studies)	no serious risk of bias	no serious inconsistency	no serious indirectness	very serious[1]	undetected	⊕⊕⊝⊝ **LOW**[1] due to imprecision	4/861 (0.5%)	7/1873 (0.4%)	**OR 0.8** (0.22 to 2.92)	**Study population**	
										**5 per 1000**	**1 fewer per 1000** (from 4 fewer to 9 more)
										**Moderate**	
										**3 per 1000**	**1 fewer per 1000** (from 2 fewer to 6 more)
**Anti-IL-17 agents subgroup vs. Placebo - Ixekizumab vs. Placebo**											
3410 (3 studies)	no serious risk of bias	no serious inconsistency	no serious indirectness	very serious[1]	undetected	⊕⊕⊝⊝ **LOW**[1] due to imprecision	3/892 (0.3%)	14/2518 (0.6%)	**OR 1.61** (0.54 to 4.76)	**Study population**	
										**3 per 1000**	**2 more per 1000** (from 2 fewer to 12 more)
										**Moderate**	
										**0 per 1000**	-

Anti-IL-17 agents subgroup vs. Placebo - Brodalumab vs. Placebo											
4109<br>(5 studies)	no serious risk of bias	no serious inconsistency	no serious indirectness	very serious[1]	undetected	⊕⊕⊝⊝<br>**LOW**[1]<br>due to imprecision	2/920<br>(0.2%)	15/3189<br>(0.5%)	**OR 1.89**<br>(0.6 to 5.93)	**Study population**	
										**2 per 1000**	**2 more per 1000**<br>(from 1 fewer to 11 more)
										**Moderate**	
										**0 per 1000**	-

[1] Very serious imprecision-lower boundary indicates appreciable benefit while upper boundary indicates appreciable harm.

(d) Anti-IL-12/23 agents vs. Placebo

Anti-IL-12/23 agents vs. placebo - Serious infection											
Quality assessment							Summary of Findings				
**Participants (studies) Follow up**	**Risk of bias**	**Inconsistency**	**Indirectness**	**Imprecision**	**Publication bias**	**Overall quality of evidence**	**Study event rates (%)**		**Relative effect** (95% CI)	**Anticipated absolute effects**	
							**With Placebo**	**With Anti-IL-12/23 agents**		**Risk with Placebo**	**Risk difference with Anti-IL-12/23 agents** (95% CI)
**Anti-IL-12/23 agents vs Placebo**											
5972<br>(13 studies)	serious[1]	no serious inconsistency	no serious indirectness	very serious[2]	undetected	⊕⊝⊝⊝<br>**VERY LOW**[1,2]<br>due to risk of bias, imprecision	7/2289<br>(0.3%)	15/3683<br>(0.4%)	**OR 1.33**<br>(0.55 to 3.23)	**Study population**	
										**3 per 1000**	**1 more per 1000**<br>(from 1 fewer to 7 more)
										**Moderate**	
										**0 per 1000**	-

**Anti-IL-12/23 agents vs Placebo - Ustekinumab vs Placebo**											
4477 (10 studies)	serious[1]	no serious inconsistency	no serious indirectness	very serious[2]	undetected	⊕⊝⊝⊝ **VERY LOW**[1,2] due to risk of bias, imprecision	6/1825 (0.3%)	12/2652 (0.5%)	**OR 1.4** (0.54 to 3.65)	**Study population**	
										**3 per 1000**	**1 more per 1000** (from 2 fewer to 9 more)
										**Moderate**	
										**2 per 1000**	**1 more per 1000** (from 1 fewer to 5 more)
**Anti-IL-12/23 agents vs Placebo - Guselkumab vs Placebo**											
1495 (3 studies)	no serious risk of bias	no serious inconsistency	no serious indirectness	serious[2]	undetected	⊕⊕⊕⊝ **MODERATE**[2] due to imprecision	1/464 (0.2%)	3/1031 (0.3%)	**OR 1.01** (0.1 to 10.09)	**Study population**	
										**2 per 1000**	**0 more per 1000** (from 2 fewer to 19 more)
										**Moderate**	
										**0 per 1000**	**-**

[1] Information from studies at high risk of bias.
[2] Very serious imprecision-lower boundary indicates appreciable benefit while upper boundary indicates appreciable harm

(e) Anti-IL-17 agents vs. TNFi

Anti-IL-17 agents vs. TNFi - Serious infection											
**Quality assessment**							**Summary of Findings**				
**Participants (studies) Follow up**	**Risk of bias**	**Inconsistency**	**Indirectness**	**Imprecision**	**Publication bias**	**Overall quality of evidence**	**Study event rates (%)**		**Relative effect** (95% CI)	**Anticipated absolute effects**	
							**With TNFi**	**With Anti-IL-17 agents**		**Risk with TNFi**	**Risk difference with Anti-IL-17 agents** (95% CI)
**Anti-IL-17 agents vs. TNFi**											
4327 (2 studies)	no serious risk of bias	no serious inconsistency	no serious indirectness	very serious[1]	undetected	⊕⊕⊖⊖ **LOW**[1] due to imprecision	7;1063 (0.7%)	21;3264 (0.6%)	**OR 0.99** (0.42 to 2.34)	**Study population**	
										**7 per 1000**	**0 fewer per 1000** (from 4 fewer to 9 more)
										**Moderate**	
										**8 per 1000**	**0 fewer per 1000** (from 5 fewer to 11 more)

[1] Very serious imprecision-lower boundary indicates appreciable benefit while upper boundary indicates appreciable harm.

(f) Anti-IL-17 agents vs. Anti-IL-12/23 agents

Anti-IL-17 agents vs. Anti-IL-12/23 agents - Serious infection											
**Quality assessment**							**Summary of Findings**				
**Participants (studies) Follow up**	**Risk of bias**	**Inconsistency**	**Indirectness**	**Imprecision**	**Publication bias**	**Overall quality of evidence**	**Study event rates (%)**		**Relative effect** (95% CI)	**Anticipated absolute effects**	
							**With Anti-IL-12/23 agents**	**With Anti-IL-17 agents**		**Risk with Anti-IL-12/23 agents**	**Risk difference with Anti-IL-17 agents** (95% CI)
**Anti-IL-17 agents vs. Ustekinumab**											
5834 (5 studies)	no serious risk of bias	no serious inconsistency	no serious indirectness	very serious[1]	undetected	⊕⊕⊖⊖ **LOW**[1] due to imprecision	12/1992 (0.6%)	19/3842 (0.5%)	**OR 0.95** (0.44 to 2.05)	**Study population**	
										**6 per 1000**	**0 fewer per 1000** (from 3 fewer to 6 more)
										**Moderate**	
										**6 per 1000**	**0 fewer per 1000** (from 3 fewer to 6 more)

[1] Very serious imprecision-lower boundary indicates appreciable benefit while upper boundary indicates appreciable harm.

(g) Anti-IL-12/23 agents vs. TNFi

Anti-IL-12/23 agents vs. TNFi - Serious infection											
**Quality assessment**							**Summary of Findings**				
**Participants (studies) Follow up**	**Risk of bias**	**Inconsistency**	**Indirectness**	**Imprecision**	**Publication bias**	**Overall quality of evidence**	**Study event rates (%)**		**Relative effect** (95% CI)	**Anticipated absolute effects**	
							**With TNFi**	**With Anti-IL-12/23 agents**		**Risk with TNFi**	**Risk difference with Anti-IL-12/23 agents** (95% CI)
**Ustekinumab vs. Etanercept**											
2557 (4 studies)	no serious risk of bias	no serious inconsistency	no serious indirectness	very serious[1]	undetected	⊕⊕⊝⊝ **LOW**[1] due to imprecision	8/1377 (0.6%)	10/1180 (0.8%)	**OR 1.67** (0.63 to 4.42)	**Study population**	
										**6 per 1000**	**4 more per 1000** (from 2 fewer to 19 more)
										**Moderate**	
										**6 per 1000**	**4 more per 1000** (from 2 fewer to 20 more)

[1] Very serious imprecision-lower boundary indicates appreciable benefit while upper boundary indicates appreciable harm.
